# MIND versus MSN: A systematic evaluation of test–retest reliability and age sensitivity for T1-weighted structural similarity networks

**DOI:** 10.1162/NETN.a.553

**Published:** 2026-07-20

**Authors:** Jiaqi Gao, Yang Hu

**Affiliations:** Faculty of Psychology, Shandong Normal University, Jinan, China; Shandong Provincial Key Laboratory of Brain Science and Mental Health, Jinan, China; Independent Researcher

**Keywords:** MSN, MIND, Test–retest reliability, Age sensitivity, Structural similarity network, T1-weighted MRI

## Abstract

Constructing structural similarity networks from T1-weighted MRI offers a powerful means to characterize brain organization. Two prominent methods for constructing such networks, Morphometric Similarity Network (MSN) and Morphometric INverse Divergence (MIND), have been proposed. However, a systematic evaluation of the test–retest reliability and age sensitivity of both MIND and MSN is still lacking. The present study comprehensively assessed these properties to inform the reliability and validity of both approaches. Test–retest reliability was evaluated by the intraclass correlation coefficient (ICC) using two public datasets containing repeated MRI scans. Age sensitivity was examined by conducting edge-wise comparisons between younger and older age groups, as well as by training machine learning models to predict individual age, using two public lifespan datasets. Additionally, several practical variants of MIND and MSN were explored by constructing networks with different morphological feature sets. Results demonstrated that MSN exhibited higher test–retest reliability, whereas MIND showed greater age sensitivity when both methods employed the same five features. Both methods revealed distinct spatial patterns that differentiate older from younger adults. Notably, the choice of feature sets substantially influenced reliability and age sensitivity. These findings offer empirical guidance for methodological selection and highlight the importance of feature optimization in future studies.

## Author Summary

To map brain organization, researchers can construct networks based on structural similarity between regions from MRI scans. Two main methods exist, Morphometric Similarity Network (MSN) and Morphometric INverse Divergence (MIND), but their test–retest reliability and sensitivity to age-related changes have not been systematically compared. This study evaluated both properties of MIND and MSN using multiple public datasets. Results show that MSN offers higher reliability across repeated scans, whereas MIND is more sensitive to age-related changes, when both methods use the same set of morphological features. Both methods reveal distinct network patterns related to brain aging. The choice of morphological features substantially influences these results. These findings clarify the distinct strengths of each method and highlight the importance of feature selection in structural similarity network studies.

## INTRODUCTION

It is generally accepted that the brain is organized as an interconnected network and network construction is a major goal in neuroimaging data analysis. While functional and diffusion MRI have been predominantly used for network mapping, T1-weighted structural MRI could be a valuable source for characterizing brain networks. Compared with functional and diffusion MRI, T1-weighted structural MRI has much better spatial resolution, anatomical fidelity, signal-to-noise ratio, and clinical availability. Therefore, networks derived from T1-weighted MRI may provide a different view of human brain organization. Many types of approaches to build T1-weighted structural networks have been proposed (for instance, Kong et al., 2014; W. Li et al., 2017; Mechelli et al., 2005; K. Zhao et al., 2021; for reviews, see Cai et al., 2023; J. Wang & He, 2024). These approaches are grounded in the observation that spatially distinct brain regions exhibit similarity in structural properties at both the population and individual levels. Accordingly, the networks produced by these methods are also referred to as structural similarity networks. The observed interregional structural similarity likely arises from a combination of factors, including shared genetic influences, coordinated developmental processes, experience-dependent plasticity, and axonal connectivity (Alexander-Bloch et al., 2013; Evans, 2013; Sebenius et al., 2025). Despite the variety of available methods, comparative studies among them remain scarce, and optimal methodological practices are still poorly understood.

**Figure 1.**
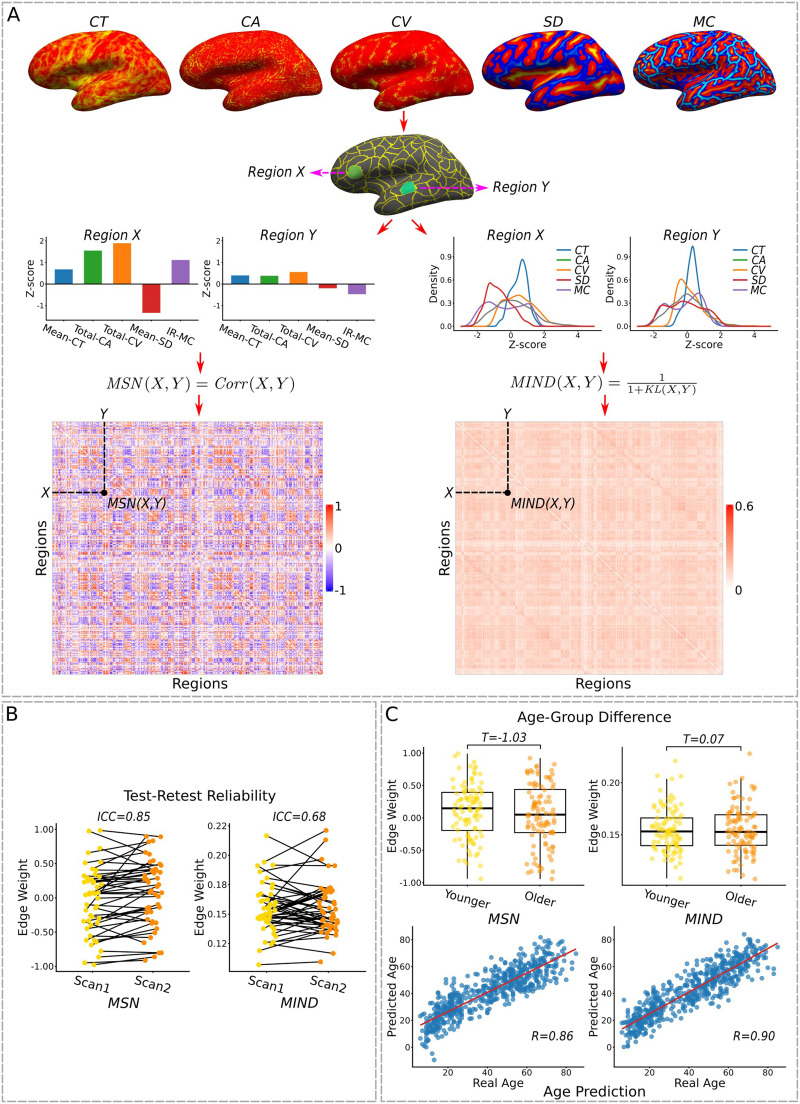
Schematic overview of the study. (A) Construction of MIND and MSN networks using five morphological features: cortical thickness (CT), cortical area (CA), cortical volume (CV), sulcal depth (SD), and mean curvature (MC). For MSN, one summary statistic per feature was derived, including mean CT, total CA, total CV, mean SD, and integrated rectified MC (IR-MC), and edge weights were defined as Pearson correlations between these summary statistics. For MIND, edge weights were calculated as the transformed KL divergence between the multivariate distributions of the five features across region pairs. (B) Test–retest reliability of each edge in MIND and MSN networks was assessed using the intraclass correlation coefficient (ICC). (C) For each edge, age-group differences (younger vs. older adults) were examined. In addition, all edges of the MIND and MSN networks were used as predictors in age-prediction models.

Of these existing approaches for constructing T1-weighted structural similarity networks, Morphometric Similarity Network (MSN; Seidlitz et al., 2018) and Morphometric INverse Divergence (MIND; Sebenius et al., 2023) have gained much attention in the field (Liang et al., 2025; Sebenius et al., 2025; J. Wang & He, 2024). Please note that the term “MSN” is used here to denote a network mapping approach, rather than the network it constructs. This usage differs from that of the original publication. In the MSN approach, each brain region was represented as a vector of summary morphological features and the edge weight was estimated as the Pearson correlation coefficient between two vectors of morphological features from two brain regions (see Figure 1A). Several lines of validation analyses have revealed that the network derived by MSN exhibits a significant correspondence with cortical cytoarchitectonic classes, gene expression, and axonal connectivity (Seidlitz et al., 2018). It should also be noted that the network derived by MSN in these biological validations was constructed using morphological features from multiple MRI modalities instead of T1-weighted MRI alone. Different from the MSN approach, the MIND approach utilized vertex-level morphological features and each brain region was modeled as a multivariate distribution. The edge weight between two brain regions was estimated as a transformed inverse of Kullback–Leibler (KL) divergence (see Figure 1A). Similar validations mentioned above were made for MIND, and the network constructed by MIND displays significantly higher biological validity than the MSN when both networks were based on the same five morphological features from T1-weighted MRI (Sebenius et al., 2023). In addition, the MIND outperforms the MSN when networks derived by both methods were trained for age prediction via machine learning models (Sebenius et al., 2023). In summary, there is initial evidence to show that the MIND approach is superior to the MSN approach in terms of biological validity.

The current study extends the work of Sebenius et al. (2023) by focusing on two critical aspects: test–retest reliability and age sensitivity. For a certain MRI-based brain measurement method like MIND, the test–retest reliability reflects the extent of agreement among the resulting metric values obtained from repeated MRI scans. Reliability is a prerequisite for biological validity, and an acceptably high level of test–retest reliability is also a must for clinical translation and tracking individual-level brain variability (Xu et al., 2023; Zuo et al., 2019). Currently, the test–retest reliability of MIND/MSN and the comparison between MIND and MSN in reliability are still lacking. On the other hand, age sensitivity contributes to the establishment of biological validity. As human brain networks reorganize dynamically across the entire lifespan, an accurate characterization of brain network should be sensitive to the age-related changes. In the work of Sebenius et al. (2023), the age sensitivity was assessed only in a limited age range (8–35 years), and the current study replicated the analysis using lifespan samples, ranging from childhood to late adulthood. In addition, in the work of Sebenius et al. (2023), all MIND/MSN edges as a whole were fed into machine learning models for age prediction. However, univariate analysis by treating each network edge separately is commonly adopted. Therefore, the current study also examined the difference between younger and older age groups at the edge level to assess age sensitivity. Several studies have investigated the relationship between structural similarity and age across the lifespan using MIND, MSN, or related approaches (Dorfschmidt et al., 2024; J. Li, Wang, et al., 2024; Liang et al., 2025; Ruan et al., 2023). Nonetheless, a direct comparison between MIND and MSN remains lacking. By evaluating the test–retest reliability and age sensitivity of MIND/MSN, this study aims to provide a comprehensive assessment of their respective strengths and limitations. These empirical findings will inform methodological selection for T1-weighted structural similarity network analyses in future research. An overview of the study design is presented schematically in Figure 1.

## METHODS

### Datasets

In this study, the test–retest reliability of MIND and MSN was evaluated using two public datasets with repeated scanning sessions: BNU1 and HNU1. The BNU1 dataset (Lin et al., 2015) includes 50 participants, each of whom underwent two T1-weighted MRI scans with an approximate interval of 6 weeks. The HNU1 dataset (Chen et al., 2015) includes 30 participants, each of whom underwent 10 T1-weighted MRI scans within about 1 month. To assess the age sensitivity of MIND and MSN, two additional public datasets with lifespan samples were employed: enhanced Nathan Kline Institute-Rockland Sample (eNKI) and Cambridge Centre for Ageing and Neuroscience (Cam-CAN). The eNKI dataset (Nooner et al., 2012; Tobe et al., 2022) includes 1,303 participants aged 6–85 years, each of whom had a T1-weighted structural image. The Cam-CAN dataset (Taylor et al., 2017) includes 653 participants aged 18–88 years, each of whom had a T1-weighted structural image. All T1-weighted images across these four public datasets had a whole-brain coverage and a spatial resolution of about 1 mm. For detailed MRI acquisition parameters, please refer to the relevant publications associated with each dataset. Informed consent was obtained from all participants at each original study site, in accordance with the respective institutional review boards.

### Image Preprocessing and Quality Control

Raw T1-weighted images were preprocessed using the recon-all pipeline of FreeSurfer (v6.0; Dale et al., 1999; Fischl et al., 1999). The main processing procedures included brain extraction, tissue segmentation, surface reconstruction, and calculation of morphological measures. For quality control, the raw data were visually checked to exclude participants with anatomical abnormalities and apparent artifacts. After preprocessing, the accuracy of brain extraction, tissue segmentation, and surface reconstruction were visually checked to exclude large errors. Furthermore, the Euler number generated by recon-all was employed as a quantitative measure to assess raw data quality. This measure has been demonstrated to be highly predictive of manual quality ratings (Rosen et al., 2018), with lower Euler number indicating poorer image quality. Since the Euler number is dataset-dependent and lacks an absolute quality threshold, the Euler numbers of all participants in each dataset were converted into modified *z* scores and the participants with modified *z* scores lower than −3.5 were treated as outliers and excluded from downstream analysis. The distribution of Euler numbers for each dataset is shown in Supporting Information Figure S1. After quality control, the final sample size and demographic characteristics of each dataset are summarized in Table 1.

**Table 1.** Demographic characteristics of the participants after quality control across the four datasets

Dataset	Sample size	Age (*M* ± *SD*)	Age range	Sex (F/M)
BNU1	47	23.06 ± 2.39	19–30	21/26
HNU1	24	24.46 ± 2.41	21–30	13/11
eNKI	1132	40.69 ± 20.67	6–85	710/422
Cam-CAN	591	53.05 ± 18.11	18–88	300/291

### Network Construction

To construct MIND and MSN networks, the selection of an appropriate set of morphological features and a cortical parcellation atlas is essential. Following the approach of Sebenius et al. (2023), five vertex-level morphological features including CT, CA, CV, SD, and MC were used for MIND. These features were automatically generated by FreeSurfer’s recon-all by default. For MSN, summary statistics of the five morphological features including mean CT, total CA, total CV, mean SD, and IR-MC were calculated for each cortical region. For parcellation, we adopted the publicly available DK308 atlas (Whitaker et al., 2016), which has been used in Seidlitz et al. (2018), and a symmetric version was also employed in Sebenius et al. (2023). The DK308 atlas is a subdivision version of the widely used Desikan-Killiany atlas (DK; Desikan et al., 2006), comprising 308 regions of approximately equal size. The DK atlas parcellates the cerebral cortex based on the gyral patterns. To ensure that the results were not substantially influenced by the choice of parcellation atlas, we repeated the analyses using the widely used Schaefer atlas (Schaefer et al., 2018). The Schaefer atlas provides multiresolution parcellations ranging from 100 to 1,000 regions and we selected the 300-region version (hereafter referred to as Schaefer300) to match the spatial resolution of DK308 atlas. The Schaefer atlas parcellates the cerebral cortex based on functional connectivity patterns, in contrast to the structurally defined DK308 atlas. Based on the selected morphological feature set and parcellation atlas, we calculated the MIND and MSN networks for each participant.

The selection of morphological features for constructing T1-weighted structural similarity networks remains an unresolved methodological issue. In the work of Sebenius et al. (2023), five morphological features were adopted without providing explicit justification. Moreover, different studies have employed varying sets of morphological features (J. Li, Zhang, et al., 2024; Y. Wang et al., 2024) further highlighting the lack of consensus. To investigate how the choice of feature sets influences the results, we evaluated several practical variants of both MIND and MSN. For MIND, we constructed networks using either CT or CV alone. These two features are highly prevalent in neuroimaging research, largely due to their intuitive biological interpretability. For clarity, we refer to the MIND method with all five features as MIND-5F, the version based solely on CT as MIND-CT, and the one using only CV as MIND-CV. For MSN, networks were constructed using either an expanded set of morphological features or a broader range of summary statistics. On one hand, four additional morphological features, namely, integrated rectified Gaussian curvature, mean folding index, mean intrinsic curvature index, and mean local gyrification index, were incorporated, resulting in a total of nine features for network construction. These features were also derived using FreeSurfer’s recon-all pipeline and have been commonly employed in prior studies on MSN networks (J. Li, Zhang, et al., 2024). On the other hand, beyond using a single summary statistic per feature, we computed four types of summary statistics, including mean, standard deviation, skewness, and kurtosis, for each of the five original morphological features. This yielded a total of 20 statistical measures (5 features × 4 statistics) for building the MSN network. For clarity, we denote the MSN method based on five features as MSN-5F, the version with nine features as MSN-9F, and the one incorporating five features with four statistical measures each as MSN-5F4S. In summary, this study evaluated a total of six network construction methods: the baseline MIND-5F and MSN-5F, along with several variants, MIND-CT and MIND-CV (adapted from MIND-5F), as well as MSN-9F and MSN-5F4S (adapted from MSN-5F). The code used to extract morphological features from FreeSurfer’s recon-all output and to compute both MIND and MSN networks is provided in Supporting Information Section S1. The MIND network was computed using the MIND package (https://github.com/isebenius/MIND) in Python (v3.12.4). Morphological feature extraction was carried out with the fsbrain package (v0.5.5; Schäfer & Ecker, 2020), in combination with FreeSurfer tools.

### Test–Retest Reliability Analysis

To evaluate the test–retest reliability of MIND and MSN, we calculated the ICC, the most widely adopted metric in this context, for each element of the network matrices derived by MIND and MSN. ICC quantifies the ratio of between-individual variance and total variance (the sum of within-individual and between-individual variance). A high ICC indicates that within-individual variability across repeated measurements is low relative to between-individual variability. In other words, a reliable method should produce stable results for the same individual across scans while effectively differentiating between individuals. It is worth noting that Sebenius et al. (2023) evaluated consistency across individuals, consistency across different parcellation atlases, and robustness to noisy features. However, these forms of stability are not what test–retest reliability is typically concerned with. The ICC was computed using a two-way random-effects analysis of variance model via the irr package (v0.84.1) in R (v4.5.0). Theoretically, ICC values range from 0 to 1, with higher values reflecting greater reliability. However, negative estimates may occur in practice; in such cases, these values were set to zero, consistent with conventional practice. For simplified interpretation, ICC values were categorized into three levels based on previous studies (Hu et al., 2023; Koo & Li, 2016): poor (ICC < 0.5), moderate (0.5 ≤ ICC < 0.75), and good (ICC ≥ 0.75).

The mean ICC across all matrix elements was computed to represent the overall reliability of each method. To determine whether significant differences exist in overall reliability between MIND-5F and MSN-5F, as well as between each baseline method and its respective variants, a bootstrap procedure was employed. Specifically, participants were resampled with replacement 2,000 times. For each bootstrap sample, the mean ICC was recalculated for every network. When comparing networks constructed by two methods (e.g., MIND-5F and MSN-5F), the difference in mean ICC was recorded per resample. Based on the resulting distribution of 2,000 differences, a 95% bias-corrected and accelerated (BCa) confidence interval (CI) was computed to evaluate the statistical significance of the ICC difference. A CI excluding zero indicated a statistically significant difference in mean ICC between the two methods. It is noteworthy that, although our analysis directly compared network-level summary statistics (mean ICCs), the use of identical bootstrap samples across all networks ensured that the resulting inference is mathematically equivalent to first computing edge-wise ICC differences and then testing their mean, thereby fully accounting for the paired structure of the data. To clarify whether the observed difference in mean ICC between two methods was driven by a few edges with large discrepancies or was broadly distributed across many edges, we calculated the proportion of edges on which one method yielded higher ICC values than the other.

In addition to the ICC, test–retest reliability was evaluated with the recently proposed discriminability statistic (Discr; Bridgeford et al., 2021). Discr measures the fraction of pairwise comparisons in which within-individual similarity exceeds between-individual similarity, providing a direct quantification of a method’s ability to discriminate across individuals. As Discr is rank-based, it is more robust to outliers than the ICC. Discr was computed for all network edges using the mgc package (v2.0.2) in R. Discr values range from 0 to 1, with higher values indicating better reliability. Mean Discr was also calculated to represent overall reliability, and differences in overall reliability between methods were tested using the same bootstrap procedure described earlier. To reduce computational cost, Discr analysis was restricted to the BNU1 dataset with 1,000 resampling iterations in the bootstrap. The code used for ICC and Discr computation, bootstrap analysis, and CI estimation is available in Supporting Information Section S2. It should be noted that CIs and *p* values reported in this study were not adjusted for multiple comparisons. This is due to the large number of statistical inferences involved and the practical difficulty in defining a clear “family” of tests for correction. As a result, the unadjusted intervals and *p* values are presented as they may offer a more informative view of the observed effects.

### Age Sensitivity Analysis

We evaluated the age sensitivity of MIND and MSN from two complementary perspectives. First, we selected 100 participants (50 females) aged 20–40 years as the younger adult group and another 100 participants (50 females) aged 60–80 years as the older adult group from the lifespan sample. In contrast to prior studies that treat age as a continuous variable (J. Li, Wang, et al., 2024; Ruan et al., 2023), we chose to compare average group-level effects between younger and older adults. This approach was adopted because the relationship between age and structural similarity is complex and potentially nonlinear, making such analyses susceptible to bias without a very large sample. For each of the six networks derived by MIND/MSN and their variants, edge-wise differences between the younger and older age groups were assessed using general linear models, with sex, total intracranial volume (TIV), and Euler number included as covariates. Multiple comparisons were corrected using the Benjamini-Hochberg false discovery rate (FDR) method, with statistical significance defined as *p* < 0.05 after FDR adjustment. The proportion of significant edges, calculated as the ratio of significant edges to total edges, was used to quantify overall age sensitivity. To determine whether significant differences in age sensitivity exist between MIND-5F and MSN-5F, as well as between each baseline method and its variants, we employed a bootstrap procedure. Participants within each group were independently resampled with replacement 2,000 times. For each bootstrap sample, the group comparison analysis was repeated and the proportion of significant edges was recalculated for every network. When comparing two methods (e.g., MIND-5F vs. MSN-5F), the difference in the proportion of significant edges was recorded per resample. A 95% BCa CI was then derived from the resulting distribution of 2,000 differences. A CI excluding zero indicated a statistically significant difference in the proportion of significant edges between the networks derived by two methods. In addition to significance testing, effect sizes were quantified. For each edge-wise comparison, the absolute value of Cohen’s *d* was calculated as a measure of effect size. The mean Cohen’s *d* across all edges in a network served as an overall index of age sensitivity, and differences in mean effect size between methods were evaluated using the same bootstrap approach described above. To reduce computational load, effect-size analysis was performed only on the eNKI dataset, with 1,000 resampling iterations in the bootstrap. Effect sizes were computed using the emmeans package (v1.11.2.8) in R.

Second, we employed a linear-kernel support vector regression (SVR) model to predict individual age. A fivefold cross-validation framework was adopted, where each fold served once as the test set while the remaining four were used for training. An inner fivefold cross-validation was applied during training for hyperparameter tuning. All edges of each network were used as predictors. To evaluate prediction accuracy, we computed the partial Pearson correlation between predicted and actual age, controlling for sex, TIV, and Euler number (Dinga et al., 2020). In addition to partial Pearson correlation, partial Spearman correlation and mean absolute error between predicted and true age were also computed as alternative accuracy metrics, with the same covariates. To examine potential sex differences in prediction performance, accuracy metrics were also calculated separately for female and male participants during model testing. To account for randomness in data partitioning, the entire training–testing procedure was repeated four times with different random seeds, yielding 20 accuracy estimates in total (5 folds × 4 repetitions). To assess whether differences in age prediction accuracy between MIND-5F and MSN-5F, or between each baseline method and its variants, were statistically significant, paired *t* tests were conducted with a significance threshold of *p* < 0.05. SVR modeling was implemented using the e1071 (v1.7.14), caret (v7.0.1), foreach (v1.5.2), doParallel (v1.0.17), and doRNG (v1.8.6.2) packages. The code used for group comparisons, bootstrap analysis, and age prediction is provided in Supporting Information Section S3. The ggplot2 (v3.5.2), ggbeeswarm (v0.7.3), and ComplexHeatmap (v2.22.0; Gu, 2022) packages were used for visualization.

## RESULTS

**Figure 2.**
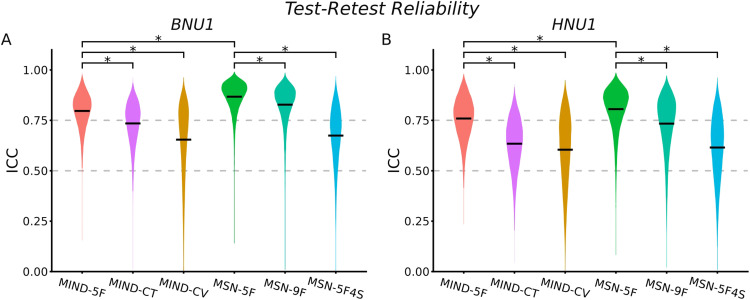
Violin plots of edge-wise ICCs for networks derived by MIND-5F, MSN-5F, and their variants using the DK308 atlas in (A) the BNU1 dataset and (B) the HNU1 dataset. Statistical differences in mean ICCs between networks derived by MIND-5F and MSN-5F, as well as between networks derived by MIND-5F/MSN-5F and their respective variants, were assessed via bootstrapping. The horizontal black bar within each violin represents the mean ICC for the corresponding network. Asterisks indicate statistical significance at an alpha level of 0.05.

As shown in Figure 2 and Table 2, both MIND-5F and MSN-5F exhibited good overall test–retest reliability, though MSN-5F demonstrated significantly higher reliability than MIND-5F. Although the difference in mean edge-wise ICC between the two methods was relatively modest, the advantage of MSN-5F was widespread at the level of individual edges, with approximately 70%–80% of edges derived from MSN-5F showing higher ICC values than those derived from MIND-5F. Comparisons between each baseline method and its variants revealed that MIND-CT and MIND-CV showed only moderate reliability, which was significantly lower than that of MIND-5F. Similarly, MSN-9F and MSN-5F4S displayed moderate-to-good reliability but were also significantly lower than MSN-5F. These findings were consistent across different datasets and parcellation atlases (see Supporting Information Figure S2 and Table S1). ICC matrices for all six networks are provided in Supporting Information Figures S3–S6. A similar pattern of results was observed when test–retest reliability was assessed using the Discr (see Supporting Information Figure S7 and Table S2).

**Table 2.** Differences in mean edge-wise ICCs between networks derived by MIND-5F and MSN-5F, and between networks derived by each baseline method and its variants using the DK308 atlas

Dataset	Comparison	Difference (95% CI)	Higher-proportion
BNU1	MIND-5F vs. MSN-5F	−0.071 (−0.090, −0.054)	0.208
	MIND-5F vs. MIND-CT	0.062 (0.035, 0.074)	0.709
	MIND-5F vs. MIND-CV	0.142 (0.113, 0.156)	0.790
	MSN-5F vs. MSN-9F	0.040 (0.025, 0.047)	0.752
	MSN-5F vs. MSN-5F4S	0.193 (0.166, 0.204)	0.956
HNU1	MIND-5F vs. MSN-5F	−0.047 (−0.052, −0.037)	0.314
	MIND-5F vs. MIND-CT	0.125 (0.116, 0.132)	0.843
	MIND-5F vs. MIND-CV	0.155 (0.145, 0.158)	0.836
	MSN-5F vs. MSN-9F	0.072 (0.065, 0.077)	0.866
	MSN-5F vs. MSN-5F4S	0.190 (0.185, 0.194)	0.960

*Note:* The *Difference* column indicates the difference in mean edge-wise ICC between the two networks derived by the methods specified in the *Comparison* column. A positive difference means that the network derived by the first method listed has a higher mean ICC than the network derived by the second method. The *Higher-proportion* column shows the proportion of edges for which the first method yields higher ICCs than the second method. A proportion greater than 0.5 means that the first method demonstrated higher ICCs than the second method on the majority of edges.

**Figure 3.**
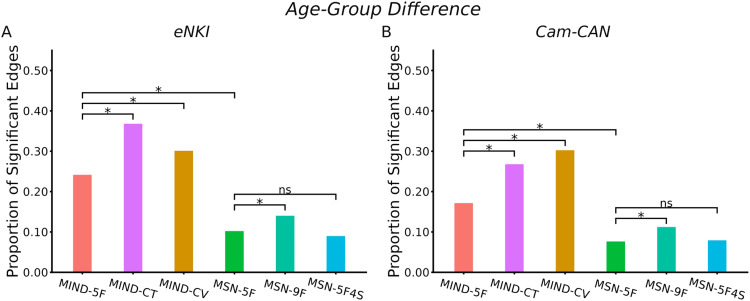
Proportions of significant edges comparing younger and older age groups for networks derived by MIND-5F, MSN-5F, and their variants using the DK308 atlas in (A) the eNKI dataset and (B) the Cam-CAN dataset. Statistical differences in these proportions between networks derived by MIND-5F and MSN-5F, as well as between networks derived by MIND-5F/MSN-5F and their respective variants, were assessed via bootstrapping. Asterisks indicate statistical significance at an alpha level of 0.05, while “ns” denotes nonsignificance.

As shown in Figure 3 and Table 3, the age-group difference analysis indicated that MIND-5F detected a significantly larger proportion of significant edges than MSN-5F, reflecting its superior age sensitivity. When comparing each baseline method with its variants, MIND-CT and MIND-CV consistently exhibited higher age sensitivity than MIND-5F across different datasets and parcellation atlases. Among MSN variants, MSN-9F showed significantly greater sensitivity than MSN-5F, whereas MSN-5F4S did not differ significantly from MSN-5F in most dataset-atlas combinations (see Supporting Information Figure S8 and Table S3). A similar pattern of results was observed when age sensitivity was quantified using Cohen’s *d* (see Supporting Information Figure S9 and Table S4).

**Table 3.** Differences in the proportion of significant edges between networks derived by MIND-5F and MSN-5F, and between each baseline method and its variants using the DK308 atlas

Dataset	Comparison	Difference (95% CI)
eNKI	MIND-5F vs. MSN-5F	0.139 (0.132, 0.170)
	MIND-5F vs. MIND-CT	−0.126 (−0.152, −0.120)
	MIND-5F vs. MIND-CV	−0.060 (−0.107, −0.037)
	MSN-5F vs. MSN-9F	−0.038 (−0.061, −0.030)
	MSN-5F vs. MSN-5F4S	0.012 (−0.016, 0.034)
Cam-CAN	MIND-5F vs. MSN-5F	0.095 (0.074, 0.140)
	MIND-5F vs. MIND-CT	−0.096 (−0.129, −0.083)
	MIND-5F vs. MIND-CV	−0.131 (−0.166, −0.117)
	MSN-5F vs. MSN-9F	−0.036 (−0.063, −0.022)
	MSN-5F vs. MSN-5F4S	−0.003 (−0.033, 0.018)

*Note:* The *Difference* column indicates the difference in proportion of significant edges between the two networks derived by the methods specified in the *Comparison* column. A positive difference means that the network derived by the first method listed has a higher proportion of significant edges than the network derived by the second method.

**Figure 4.**
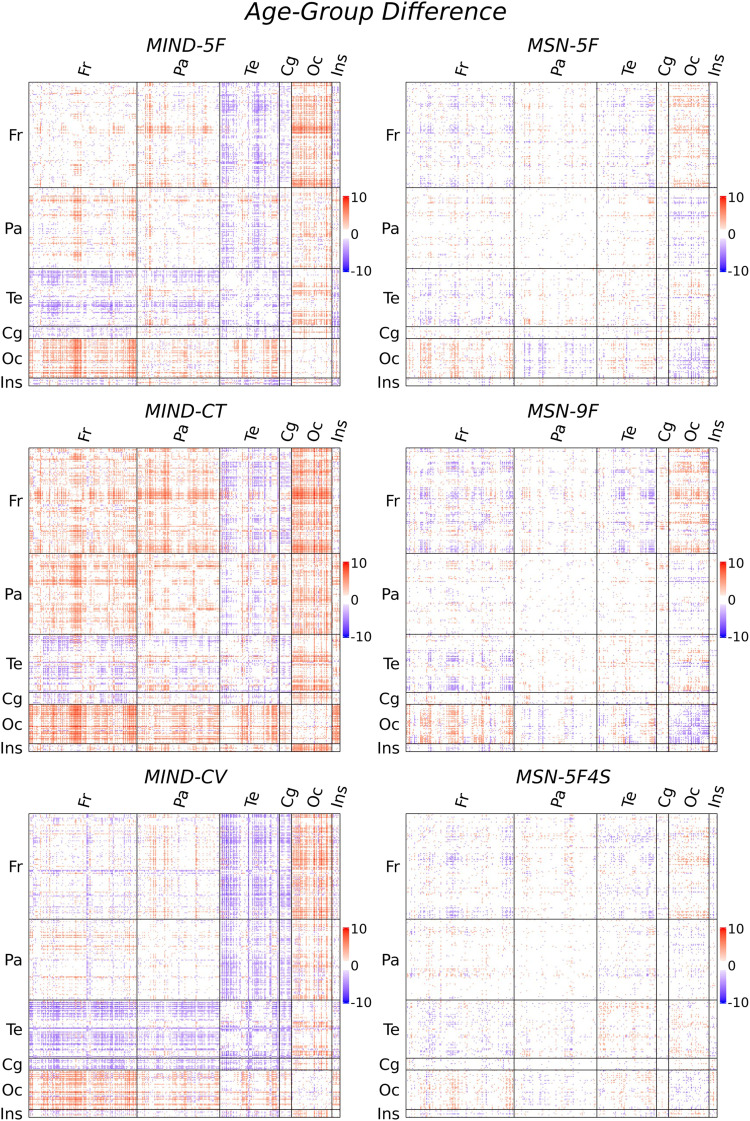
T-statistic matrices comparing younger and older age groups for networks derived by MIND-5F, MSN-5F, and their variants using the DK308 atlas in the eNKI dataset. Positive t-statistic values indicate higher edge weights in the older group compared to the younger group; negative values indicate the opposite. Nonsignificant matrix entries are set to zero. Rows and columns are clustered according to the lobar location of each brain region. Abbreviations: Fr, frontal lobe; Pa, parietal lobe; Te, temporal lobe; Cg, cingulate cortex; Oc, occipital lobe; Ins, insula.

Beyond overall sensitivity in detecting age-group differences, MIND and MSN exhibited distinct spatial patterns of these differences, as illustrated in Figure 4 and Supporting Information Figures S10–S12. For example, using MIND-5F, older adults displayed decreased connections between the temporal lobe and nonoccipital regions, alongside increased connections linking the occipital lobe to other brain regions, relative to younger adults. Using MSN-5F, by contrast, older adults showed reduced within-occipital connections but stronger fronto-occipital connections. These characteristic spatial patterns were generally maintained across the baseline methods and their respective variants, and the findings proved consistent over different datasets and parcellation atlases.

**Figure 5.**
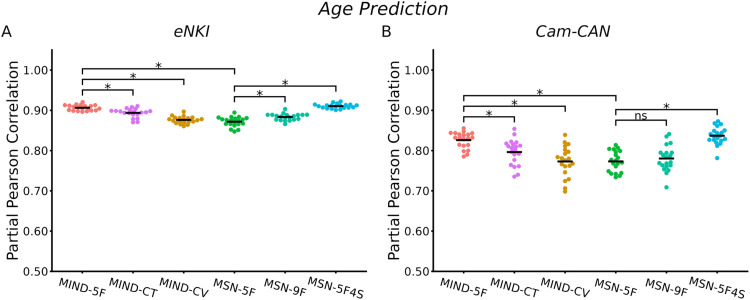
Age prediction performance of models trained on networks derived by MIND-5F, MSN-5F, and their variants using the DK308 atlas in (A) the eNKI dataset and (B) the Cam-CAN dataset. A fivefold cross-validation procedure was repeated four times to yield 20 accuracy estimates, each represented by a point in the scatter plot. In each fold, prediction accuracy was quantified by the partial Pearson correlation between predicted and true age, with sex, TIV, and Euler number as covariates. Statistical differences in prediction accuracy between networks derived by MIND-5F and MSN-5F, as well as between networks derived by MIND-5F/MSN-5F and their respective variants, were assessed using paired *t* tests. Asterisks indicate statistical significance at an alpha level of 0.05, while “ns” denotes nonsignificance.

As shown in Figure 5 and Table 4, MIND-5F achieved significantly higher age prediction accuracy, measured by partial Pearson correlation, than MSN-5F, further supporting its superior age sensitivity. Comparisons among MIND variants showed that MIND-CT and MIND-CV yielded significantly lower accuracy than MIND-5F across datasets and parcellation atlases. Among MSN variants, MSN-9F outperformed MSN-5F only in the eNKI dataset, whereas MSN-5F4S showed significantly higher accuracy than the baseline in both the eNKI and Cam-CAN datasets (see Supporting Information Figure S13 and Table S5). Similar patterns of results were observed when alternative accuracy metrics including partial Spearman correlation and mean absolute error were considered (see Supporting Information Figures S14–S15 and Tables S6–S7). Overall, age-prediction performance was comparable between female and male participants (see Supporting Information Figures S16–S19 and Tables S8–S11). Notably, although not a primary focus of this study, MSN-5F4S appeared to outperform MIND-5F. Further statistical analysis confirmed that MSN-5F4S performed significantly better than MIND-5F when using the DK308 atlas, whereas no significant difference was observed with the Schaefer300 atlas (see Supporting Information Table S12).

**Table 4.** Differences in age prediction accuracy quantified by partial Pearson correlation between MIND-5F and MSN-5F, and between each baseline method and its variants using the DK308 atlas

Dataset	Comparison	Difference (*M* ± *SD*)	T-statistic	*p* value
eNKI	MIND-5F vs. MSN-5F	0.035 ± 0.012	12.683	<0.001*
	MIND-5F vs. MIND-CT	0.013 ± 0.010	5.811	<0.001*
	MIND-5F vs. MIND-CV	0.030 ± 0.013	10.415	<0.001*
	MSN-5F vs. MSN-9F	−0.012 ± 0.010	−5.508	<0.001*
	MSN-5F vs. MSN-5F4S	−0.039 ± 0.013	−13.564	<0.001*
Cam-CAN	MIND-5F vs. MSN-5F	0.053 ± 0.023	10.411	<0.001*
	MIND-5F vs. MIND-CT	0.030 ± 0.022	6.137	<0.001*
	MIND-5F vs. MIND-CV	0.053 ± 0.029	8.108	<0.001*
	MSN-5F vs. MSN-9F	−0.007 ± 0.031	−1.050	0.307
	MSN-5F vs. MSN-5F4S	−0.064 ± 0.025	−11.615	<0.001*

*Note:* The *Difference* column reports the mean difference in partial Pearson correlation (aggregated across all cross-validation folds) between networks derived by the two methods specified in the *Comparison* column. A positive difference indicates that the network obtained with the first method exhibits higher predictive accuracy than that obtained with the second method. Asterisks in the *p value* column denote statistical significance at an alpha level of 0.05.

## DISCUSSION

This study presents a comprehensive empirical comparison of MSN and MIND in terms of two key biometric properties: test–retest reliability and age sensitivity. Three main findings emerged. First, when based on the same set of five morphological features, MSN showed higher overall test–retest reliability than MIND, whereas MIND exhibited greater age sensitivity. Second, comparisons between the baseline methods and their variants revealed considerable variations in both reliability and age sensitivity, underscoring the substantial impact of feature selection. Third, the spatial patterns of age-group differences diverged between networks derived by MIND and MSN, implying distinct neurobiological underpinnings. These findings were consistently reproduced across different datasets and parcellation atlases.

In terms of test–retest reliability, both MIND and MSN demonstrated moderate to good overall reliability, consistent with prior studies employing similar network construction approaches (W. Li et al., 2017; H. Wang et al., 2016; Y. Wang et al., 2024). More notably, numerical comparisons with functional and diffusion MRI-based networks (Buimer et al., 2020; Hu et al., 2023; T. Zhao et al., 2015) suggests that T1-weighted structural similarity networks derived by MIND and MSN probably offer superior measurement stability. Consequently, for research designs where measurement stability is critical, such as detecting longitudinal changes, MIND and MSN present a compelling alternative. Furthermore, while MSN-5F exhibited significantly higher reliability than MIND-5F, the effect size of this difference was smaller than those observed between the baseline methods (MIND-5F/MSN-5F) and their respective variants. This indicates that the choice of morphological feature set has a greater impact on reliability than the choice of network construction strategy (i.e., MIND vs. MSN).

As for age sensitivity, MIND-5F demonstrated clear advantages over MSN-5F in both age-group discrimination and individual age prediction, replicating and extending the core finding of Sebenius et al. (2023). Notably, MSN-5F4S achieved age prediction performance comparable to that of MIND-5F, suggesting that the age sensitivity of the MSN framework can be substantially enhanced through appropriate feature selection. Comparisons among MIND variants revealed that both MIND-CT and MIND-CV exhibited a marked increase in detecting group differences relative to MIND-5F. Given that CT and CV are sensitive biomarkers in both normal and pathological aging (Hu et al., 2023; Schwarz et al., 2016), networks relying solely on these features may capture more locally pronounced age-related differences. In contrast, when predicting individual age, both MIND-CT and MIND-CV underperformed compared to MIND-5F, likely because machine learning models benefit from the richer information provided by multiple complementary features. Since brain networks are multivariate in nature and predictive modeling integrates information across all edges, age-prediction performance could better reflect the overall sensitivity of a network to age. Thus, MIND-5F demonstrates higher age sensitivity at the whole-network level. However, MIND-CT and MIND-CV may be advantageous when analyzing each network edge independently. Among MSN variants, MSN-9F showed improved age sensitivity in group comparison task, while MSN-5F4S consistently outperformed MSN-5F in age prediction. Together, these findings suggest that the optimal feature set for constructing MIND and MSN networks depends on the specific research objective, whether detecting local group differences or performing individual-level prediction, as well as the underlying neural mechanisms of interest.

Beyond differences in overall age sensitivity, we also identified distinct and stable spatial patterns of age-related changes between networks derived by MIND and MSN. In general, relative to younger adults, older adults exhibited decreased connections between the temporal lobe and nonoccipital regions, alongside increased connections between the occipital lobe and other regions in MIND networks. In contrast, MSN networks in older adults showed reduced within-occipital lobe connections but enhanced fronto-occipital connections compared to younger adults. These patterns are neurobiologically plausible: the temporal lobe is known to be particularly vulnerable to aging and Alzheimer’s disease (Lockhart & DeCarli, 2014; Schwarz et al., 2016), and the inferior fronto-occipital fasciculus, which links occipital and frontal regions, has also been implicated in both aging and Alzheimer’s pathology (Jin et al., 2017; Peter et al., 2025). Notably, prior studies using either MIND or MSN have similarly reported increased structural similarity in paralimbic regions during adolescence (Dorfschmidt et al., 2024; Liang et al., 2025). Collectively, these results suggest that MIND and MSN networks capture distinct yet complementary neurobiological signatures of brain development and aging.

We observed divergent trends between MIND-5F and MSN-5F in terms of test–retest reliability and age sensitivity: MSN-5F exhibited higher reliability but lower age sensitivity. This contrast likely stems from the fact that the MSN method relies on region-wise summary statistics of morphological features, which may reduce measurement noise and thus enhance reliability, but could also attenuate subtle, biologically meaningful variations associated with age. This interpretation is partly supported by the improved age prediction performance of MSN-5F4S, which incorporates a broader set of summary statistics per feature. These findings underscore the limitation of relying solely on test–retest reliability, as high reliability can arise from stable artifacts or variations of no interest. At the same time, sensitivity to age does not ensure sensitivity to other biologically relevant factors such as cognition or neuropathology. For instance, prior study has shown that while brain structure is highly sensitive to age, it is considerably less sensitive to psychological traits (Kharabian Masouleh et al., 2019). Therefore, further comparative evaluations of MIND and MSN from multiple perspectives are needed to fully elucidate their respective strengths and limitations.

Several limitations of this study should be acknowledged, along with potential directions for future research. First, while we employed two widely used structural and functional parcellation atlases to enhance the robustness of our findings, the influence of atlas selection on network reliability and age sensitivity remains to be systematically evaluated. Second, we examined only two practical variants each for MIND and MSN; the substantial impact of feature set selection on both reliability and sensitivity underscores the need for a more comprehensive evaluation and optimization of feature choices in T1-weighted structural similarity network construction. Third, we observed distinct age-related spatial patterns in networks derived by MIND and MSN; however, the neurobiological meaning of these differences is unclear. This ambiguity underscores the need for continued efforts to clarify the biological underpinnings of networks derived by two methods. Finally, the current MIND and MSN frameworks are confined to cortical regions; extending these methods to incorporate subcortical structures represents an important direction for future methodological development.

## CONCLUSION

This study demonstrates that, when constructed using the same set of five morphological features, MIND exhibits lower test–retest reliability yet greater age sensitivity compared to MSN. Furthermore, the choice of feature set significantly influences both the reliability and age sensitivity of both methods. Distinct age-related spatial patterns were also observed between the networks derived by MIND and MSN. Together, these findings offer empirical guidance for methodological selection based on research objectives and highlight the importance of feature selection in future methodological optimization.

## ACKNOWLEDGMENTS

This work was supported by the Shandong Provincial Natural Science Foundation (Youth Program ZR2023QC226) and Youth Innovation Team Program of Shandong Universities (2022KJ252). We sincerely thank the reviewers for their time and constructive comments, which have helped us significantly improve the manuscript.

## SUPPORTING INFORMATION

Supporting information for this article is available at https://doi.org/10.1162/NETN.a.553.

## AUTHOR CONTRIBUTIONS

Jiaqi Gao: Data curation; Funding acquisition; Methodology; Writing – original draft; Writing – review & editing. Yang Hu: Conceptualization; Data curation; Formal analysis; Methodology; Writing – original draft; Writing – review & editing.

## FUNDING INFORMATION

Jiaqi Gao, Shandong Provincial Natural Science Foundation, Award ID: Youth Program ZR2023QC226. Jiaqi Gao, Youth Innovation Team Program of Shandong Universities, Award ID: 2022KJ252.

## DATA AND CODE AVAILABILITY

All MRI datasets are publicly available. The BNU1 and HNU1 datasets are available at https://fcon_1000.projects.nitrc.org/indi/CoRR/html/index.html, the eNKI dataset is available at https://fcon_1000.projects.nitrc.org/indi/enhanced/index.html, and the Cam-CAN dataset is available at https://cam-can.mrc-cbu.cam.ac.uk/dataset/.

The core code necessary to replicate the key data analyses is provided in the Supporting Information. The original scripts used to generate the results reported in this study are publicly available in the following GitHub repository: https://github.com/younghoo/paper-scripts/tree/main/Gao2025_MINDvsMSN.

## Supplementary Material



## TECHNICAL TERMS

T1-weighted MRI: A type of magnetic resonance imaging scan that creates a detailed picture of the brain’s anatomy and structure.
Structural similarity network: A network model where connections between brain regions are defined by the similarity of their structural characteristics.
Morphological features: Computational measures that quantify brain shape and structure, such as cortical thickness and surface area.
Edge weight: A numerical value assigned to a connection in a brain network, quantifying the strength of relationship between two regions.
Vertex: A basic point or building block in the 3D mesh that represents the reconstructed surface of the brain’s cortex.
Biological validity: The extent to which a computational model or measurement aligns with known facts about brain biology.
Test–retest reliability: The consistency of measurement results when the same test or analysis is applied to the same participant on multiple occasions.
Age sensitivity: The extent to which a measurement or method can detect systematic changes that are correlated with age.
Parcellation atlas: A standardized map that segments the brain into distinct regions based on specific anatomical or functional criteria.
Bootstrap: A statistical method that estimates the confidence of a result by repeatedly drawing random samples from the original data.
