# Supplementary material for: MIND versus MSN: A systematic evaluation of test–retest reliability and age sensitivity for T1-weighted structural similarity networks
Source: Netw Neurosci. 2026 Jul 20;10(3):613–29. doi: 10.1162/NETN.a.553 (PMC13418519; doi:10.1162/NETN.a.553)
Supplement: Supplementary file 1 [file netn-10-3-613-s001.pdf]

Supplementary Materials

S1. The code for extracting morphological features and computing MIND and MSN networks

```
## Note: The code below includes only core steps for clarity and ease of understanding.
## Step 1: Run FreeSurfer's recon-all pipeline to process T1-weighted images in Bash
## ${T1} represents the path to the raw T1-weighted image
## ${SUBJECT} is the subject output folder name
recon-all -i ${T1} -s ${SUBJECT} -autorecon-all

## Step 2: Transform DK308 atlas to native surface space
## ${hemi} indicates hemisphere: 'lh' for left or 'rh' for right
## ${hemi}.500.aparc.annot is the DK308 atlas annotation file
mri_surf2surf --srcsubject fsaverage --trgsubject $SUBJECT --hemi $hemi --sval-annot ${hemi}.500.aparc.annot --tval ${SUBJECTS_DIR}/${SUBJECT}/label/${hemi}.500.aparc.annot

## Step 3: Compute FreeSurfer's default summary statistics based on the DK308 atlas
 mris_anatomical_stats -mgz -cortex ${SUBJECTS_DIR}/${SUBJECT}/label/${hemi}.cortex.label -f ${SUBJECTS_DIR}/${SUBJECT}/stats/${hemi}.500.aparc.stats -b -a ${SUBJECTS_DIR}/${SUBJECT}/label/${hemi}.500.aparc.annot ${SUBJECT} ${hemi} white

## Step 4: Export summary statistics into table files for each morphological feature
for curr_meas in thickness area volume meancurv gauscurv foldind curvind
do
    aparcstats2table --subjects ${SUBJECT} --hemi ${hemi} --parc 500.aparc --meas ${curr_meas} --tablefile ${SUBJECT}_500.aparc_${curr_meas}_${hemi}.txt
done

## Step 5: Compute mean sulcal depth based on the DK308 atlas
mris_anatomical_stats -mgz -cortex ${SUBJECTS_DIR}/${SUBJECT}/label/${hemi}.cortex.label -f ${SUBJECTS_DIR}/${SUBJECT}/stats/${hemi}.500.aparc_sulc.stats -b -a ${SUBJECTS_DIR}/${SUBJECT}/label/${hemi}.500.aparc.annot -t sulc ${SUBJECT} ${hemi} white

## Step 6: Export mean sulcal depth values to a table file
aparcstats2table --subjects ${SUBJECT} --hemi ${hemi} --parc 500.aparc_sulc --meas thickness --tablefile ${SUBJECT}_500.aparc_sulc_${hemi}.txt
```

```
## Step 7: Compute mean local gyrification index using the DK308 atlas
recon-all -s ${SUBJECT} -localGI
mris_anatomical_stats -mgz -cortex
${SUBJECTS_DIR}/${SUBJECT}/label/${hemi}.cortex.label -f
${SUBJECTS_DIR}/${SUBJECT}/stats/${hemi}.500.aparc_lgi.stats -b -a
${SUBJECTS_DIR}/${SUBJECT}/label/${hemi}.500.aparc.annot -t pial_lgi
${SUBJECT} ${hemi} white

## Step 8: Export mean local gyrification index to a table file
aparcstats2table --subjects ${SUBJECT} --hemi ${hemi} --parc 500.aparc_lgi --meas
thickness --tablefile ${SUBJECT}_500.aparc_lgi_${hemi}.txt

## Step 9: Compute MIND-5F, MIND-CT and MIND-CV networks in Python
## subj_dir: absolute path to the recon-all output directory for a subject
import sys
sys.path.insert(1, '/home/huyang/Software/MIND')
MIND_5F = compute_MIND(subj_dir, ['CT','MC','Vol','SD','SA'], '500.aparc')
MIND_CT = compute_MIND(subj_dir, ['CT'], '500.aparc', resample=True)
MIND_CV = compute_MIND(subj_dir, ['Vol'], '500.aparc', resample=True)

## Step 10: Extract four summary statistics for five morphological features in R
## proj_dir: parent directory containing subject folders
## curr_sub: subject folder name with recon-all output
## curr_meas: morphological feature (e.g., 'thickness')
## curr_hemi: hemisphere ('lh' or 'rh')
## curr_func: summary function (e.g., mean, sd, skewness, kurtosis)
library(fsbrain)
morph_dat <- subject.morph.native(proj_dir, curr_sub, curr_meas, curr_hemi)
annot_dat <- subject.annot(proj_dir, curr_sub, curr_hemi, '500.aparc')
curr_dat <- data.frame(morph = morph_dat, label = annot_dat$label_names)
stat_dat <- aggregate(curr_dat$morph, list(curr_dat$label), curr_func)

## Step 11: Z-score normalization of morphological data across regions for each
subject
## subj_dat: a data.frame where rows represent features and columns represent
regions
zscore_dat <- t(scale(t(as.matrix(subj_dat)), center = TRUE, scale = TRUE))

## Step 12: Compute MSN network for a subject using Z-scored data
## zscore_dat: a data.frame where rows are features and columns are regions
MSN_dat <- cor(zscore_dat, method = 'pearson')
```

S2. The code used for ICC and Discr computation, bootstrap analysis, and confidence interval estimation

```
## Step 1: Compute edge-wise ICC and mean ICC in R
## curr_edge_mat: a matrix where rows represent subjects and columns represent
sessions
library(irr)
ICC_dat <- icc(curr_edge_mat, model = "twoway", type = "agreement", unit =
"single")
ICC_dat <- ifelse(ICC_dat$value < 0, 0, ICC_dat$value)
## all_ICC_dat: a vector containing ICC values for all unique edges
## mean_ICC: the mean ICC across all edges, representing the overall reliability of a
network
mean_ICC <- mean(all_ICC_dat)

## Step 2: Bootstrap procedure for a single edge
## NBOOT: the number of bootstrap resamplings
## NSUB: the number of subjects
NBOOT <- 2000
NSUB <- nrow(curr_edge_mat)
set.seed(100)
boot_samples <- replicate(NBOOT, sample(1:NSUB, NSUB, replace=TRUE))
# boot_ICC_dat: a vector to store bootstrap ICC values for one edge
boot_ICC_dat <- matrix(0, nrow=NBOOT, ncol=1)
for (boot_idx in c(1:NBOOT)){
    # Resample the edge data with replacement
    curr_boot_mat <- curr_edge_mat[boot_samples[,boot_idx], ]
    ## Compute ICC for the bootstrap sample
    ICC_dat <- icc(curr_boot_mat, model = "twoway", type = "agreement", unit =
"single")
    boot_ICC_dat[boot_idx,] <- ifelse(ICC_dat$value < 0, 0, ICC_dat$value)
}

## Step3: Jackknife procedure for a single edge
## jack_ICC_dat: a vector to store jackknife ICC values for one edge
jack_ICC_dat <- matrix(0, nrow=NSUB, ncol=1)
for (sub_idx in c(1:NSUB)){
    ## Leave one subject out
    curr_jack_mat <- curr_edge_mat[-sub_idx, ]
    ## Compute ICC for the jackknife sample
    ICC_dat <- icc(curr_jack_mat, model = "twoway", type = "agreement", unit =
"single")
    jack_ICC_dat[sub_idx, ] <- ifelse(ICC_dat$value < 0, 0, ICC_dat$value)
```

```
}

## Step 4: Compute 95% confidence interval using the BCa method
## curr_diff_real: observed mean ICC difference between two networks
## curr_diff_boot: vector of bootstrap mean ICC differences between two networks
## curr_diff_jack: vector of jackknife mean ICC differences between two networks
alpha <- 0.05
z0 <- qnorm(mean(curr_diff_boot < curr_diff_real))
U <- (curr_diff_real - curr_diff_jack)
a <- sum(U^3)/(6*sum(U^2)^(3/2))
lb <- pnorm(z0+(z0+qnorm(alpha/2))/(1-a*(z0+qnorm(alpha/2))))
ub <- pnorm(z0+(z0+qnorm(1-alpha/2))/(1-a*(z0+qnorm(1-alpha/2))))
curr_CI <- quantile(curr_diff_boot, c(lb, ub))

## Step 5: Compute edge-wise Discr in R
## curr_edge_dat: a vector of edge weights comprising data from all subjects and
sessions.
## sublist: a vector of subject IDs used to specify which sessions belong to the same
individual.
library(mgc)
Discr_dat <- discr.stat(curr_edge_dat, sublist)$discr
```

S3. The code used for group comparisons, bootstrap analysis, and age prediction

```
## Step 1: Edge-wise group comparison between younger and older age groups in R
## curr_edge_dat: data.frame containing subject data for a single edge
curr_lm_mod <- lm(Edge ~ Group + Sex + TIV + EulerNumber, data=curr_edge_dat)
curr_stat <- summary(curr_lm_mod)
curr_pval <- curr_stat$coefficients[2,4]
## Effect size estimation
library(emmeans)
EMM <- emmeans(curr_lm_mod, "Group")
curr_result <- summary(eff_size(EMM, sigma = sigma(curr_lm_mod), edf =
df.residual(curr_lm_mod)))
curr_CohenD <- abs(curr_result$effect.size)
## Apply BH-FDR correction across all edges
## all_pval: vector of raw p-values for all edges
pval_FDR <- p.adjust(all_pval, method = 'fdr')
## Compute proportion of significant edges after FDR correction
sig_prop <- mean(pval_FDR < 0.05)

## Step 2: Bootstrap procedure for group comparison
NSUB <- 200
```

```
NBOOT <- 2000
set.seed(100)
## Create bootstrap samples for younger (YA) and older (OA) groups separately
YA_sub_idx <- which(Group == 'YA')
YA_samples <- replicate(NBOOT, sample(YA_sub_idx, length(YA_sub_idx),
replace=TRUE))
OA_sub_idx <- which(Group == 'OA')
OA_samples <- replicate(NBOOT, sample(OA_sub_idx, length(OA_sub_idx),
replace=TRUE))
boot_samples <- rbind(YA_samples, OA_samples)
## boot_pval_dat: stores bootstrap p-values for a single edge
boot_pval_dat <- matrix(0, nrow=NBOOT, ncol=1)
for (boot_idx in c(1:NBOOT)){
    curr_boot_dat <- curr_edge_dat[boot_samples[,boot_idx], ]
    curr_lm_mod <- lm(Edge ~ Group + Sex + TIV + EulerNumber,
data=curr_boot_dat)
    curr_stat <- summary(curr_lm_mod)
    curr_pval <- curr_stat$coefficients[2,4]
    boot_pval_dat[boot_idx, ] <- curr_pval
}
## Compute proportion of significant edges for each bootstrap sample
## all_boot_pval: matrix where rows represent bootstrap iterations and columns
represent edges
boot_pval_FDR <- t(apply(all_boot_pval, 1, p.adjust, method = 'fdr'))
boot_sig_prop <- apply(boot_pval_FDR, 1, function(x){mean(x < 0.05)})

## Step 3: Jackknife procedure for group comparison
## jack_pval_dat: stores jackknife p-values for a single edge
jack_pval_dat <- matrix(0, nrow=NSUB, ncol=1)
for (sub_idx in c(1:NSUB)){
    curr_jack_dat <- curr_edge_dat[-sub_idx, ]
    curr_lm_mod <- lm(Edge ~ Group + Sex + TIV + EulerNumber,
data=curr_jack_dat)
    curr_stat <- summary(curr_lm_mod)
    curr_pval <- curr_stat$coefficients[2,4]
    jack_pval_dat[sub_idx, ] <- curr_pval
}
## Compute proportion of significant edges for each jackknife sample
jack_pval_FDR <- t(apply(all_jack_pval, 1, p.adjust, method = 'fdr'))
jack_sig_prop <- apply(jack_pval_FDR, 1, function(x){mean(x < 0.05)})

## Step 4: SVR modeling for age prediction
library(e1071)
```

```
library(caret)
library(foreach)
library(doParallel)
library(doRNG)
## Function to calculate accuracy metrics
calc_ACC <- function(y_real, y_predict, cov_dat){
    ## y_real: actual values of target variable
    ## y_predict: predicted values from SVR model
    ## cov_dat: data.frame of covariates
    ## Partial Pearson correlation
    y_real_resid <- resid(lm(y_real ~ ., data = cov_dat))
    y_predict_resid <- resid(lm(y_predict ~ ., data = cov_dat))
    PPC_dat <- cor(y_real_resid, y_predict_resid)
    ## Partial Spearman correlation
    cov_dat_rank <- as.data.frame(apply(cov_dat, 2, rank))
    y_real_resid <- resid(lm(rank(y_real) ~ ., data = cov_dat_rank))
    y_predict_resid <- resid(lm(rank(y_predict) ~ ., data = cov_dat_rank))
    PSC_dat <- cor(y_real_resid, y_predict_resid)
    ## Mean absolute error
    curr_lm_mod <- lm(y_real ~ ., data = cov_dat)
    y_real_adjust <- resid(curr_lm_mod) + coef(curr_lm_mod)[1]
    curr_lm_mod <- lm(y_predict ~ ., data = cov_dat)
    y_predict_adjust <- resid(curr_lm_mod) + coef(curr_lm_mod)[1]
    MAE_dat <- mean(abs(y_real_adjust - y_predict_adjust))
    ## Combine all metrics
    ACC_dat <- c(PPC_dat, PSC_dat, MAE_dat)
    return(ACC_dat)
}
## Evaluate prediction accuracy using all samples or female/male samples
SVR_ACC <- function(y_real, y_predict, cov_dat){
    ## Using all samples
    all_ACC <- calc_ACC(y_real, y_predict, cov_dat)
    ## Using female samples
    female_idx <- cov_dat$Sex == 'F'
    female_ACC <- calc_ACC(y_real[female_idx], y_predict[female_idx],
cov_dat[female_idx, -1])
    ## Using male samples
    male_idx <- cov_dat$Sex == 'M'
    male_ACC <- calc_ACC(y_real[male_idx], y_predict[male_idx], cov_dat[male_idx,
-1])
    ## Combine all metrics
    ACC_dat <- c(all_ACC, female_ACC, male_ACC)
    return(ACC_dat)
```

```
}
## Repeated K-fold cross validation function
SVR_CV <- function(x, y, z, K=5, N=4, myseed=1){
    ## x: predictor matrix (rows: observations, columns: features)
    ## y: target variable vector
    ## z: covariate data.frame (rows: observations, columns: covariates)
    ## K: number of folds in cross-validation
    ## N: number of repetitions
    ## myseed: random seed
    ## Set random seed to ensure reproducibility
    set.seed(myseed)
    ## Randomly split the data into K folds and repeat N times
    all_folds <- matrix(0, nrow=length(y), ncol=N)
    for (col_idx in c(1:N)){
        all_folds[,col_idx] <- createFolds(y, k=K, list = FALSE)
    }
    ## Loop each run of train-test procedure
    M <- N * K
    output <- foreach(curr_run = 1:M, .combine='rbind') %dorng% {
        curr_rep <- (curr_run - 1) %/% K + 1
        curr_fold <- (curr_run - 1) %% K + 1
        ## Partition data into training and testing sets
        sub_idx <- which(all_folds[, curr_rep] == curr_fold)
        x_train <- x[-sub_idx,]
        y_train <- y[-sub_idx]
        x_test <- x[sub_idx,,drop=FALSE]
        y_test <- y[sub_idx]
        z_test <- z[sub_idx,,drop=FALSE]
        ## Hyperparameter tuning with inner cross-validation
        model_tune <- tune(svm, x_train, y_train, kernel='linear',
                ranges = list(epsilon = c(0.01, 0.1, 0.5, 1), cost = c(0.1, 1, 10, 100,
1000)),
                tunecontrol = tune.control(nrepeat = 1, sampling = "cross",
cross=K))
        model_train <- model_tune$best.model
        ## Predict
        y_predict <- predict(model_train, x_test)
        ## Evaluate performance
        SVR_ACC(y_test, y_predict, z_test)
    }
    return(output)
}
```

Table S1. Differences in mean edge-wise ICCs between networks derived by MIND-5F and MSN-5F, and between networks derived by each baseline method and its variants using the Schaefer300 atlas

Dataset	Comparison	Difference (95% CI)	Higher-Proportion
BNU1	MIND-5F vs. MSN-5F	-0.026 (-0.032, -0.018)	0.373
	MIND-5F vs. MIND-CT	0.089 (0.079, 0.096)	0.793
	MIND-5F vs. MIND-CV	0.161 (0.148, 0.166)	0.849
	MSN-5F vs. MSN-9F	0.025 (0.013, 0.033)	0.676
	MSN-5F vs. MSN-5F4S	0.170 (0.144, 0.182)	0.925
HNU1	MIND-5F vs. MSN-5F	-0.011 (-0.018, -0.006)	0.437
	MIND-5F vs. MIND-CT	0.138 (0.131, 0.143)	0.859
	MIND-5F vs. MIND-CV	0.147 (0.140, 0.150)	0.837
	MSN-5F vs. MSN-9F	0.047 (0.041, 0.053)	0.779
	MSN-5F vs. MSN-5F4S	0.166 (0.157, 0.172)	0.924

Notes: The *Difference* column indicates the difference in mean edge-wise ICC between the two networks derived by the methods specified in the *Comparison* column. A positive difference means that the network derived by the first method listed has a higher mean ICC than the network derived by the second method. The *Higher-Proportion* column shows the proportion of edges for which the first method yields higher ICCs than the second method. A proportion greater than 0.5 means that the first method demonstrated higher ICCs than the second method on the majority of edges.

Table S2. Differences in mean edge-wise Discr statistics between networks derived by MIND-5F and MSN-5F, and between networks derived by each baseline method and its variants in the BNU1 dataset

Atlas	Comparison	Difference (95% CI)	Higher-Proportion
DK308	MIND-5F vs. MSN-5F	-0.055 (-0.064, -0.049)	0.152
	MIND-5F vs. MIND-CT	0.037 (0.030, 0.042)	0.728
	MIND-5F vs. MIND-CV	0.079 (0.072, 0.086)	0.825
	MSN-5F vs. MSN-9F	0.029 (0.026, 0.032)	0.816
	MSN-5F vs. MSN-5F4S	0.116 (0.112, 0.120)	0.985
Schaefer300	MIND-5F vs. MSN-5F	-0.024 (-0.028, -0.021)	0.323
	MIND-5F vs. MIND-CT	0.052 (0.050, 0.056)	0.798
	MIND-5F vs. MIND-CV	0.090 (0.087, 0.095)	0.874
	MSN-5F vs. MSN-9F	0.019 (0.016, 0.024)	0.724
	MSN-5F vs. MSN-5F4S	0.100 (0.097, 0.105)	0.963

Notes: The *Difference* column indicates the difference in mean edge-wise Discr between the two networks derived by the methods specified in the *Comparison* column. A positive difference means that the network derived by the first method listed has a higher mean Discr than the network derived by the second method. The *Higher-Proportion* column shows the proportion of edges for which the first method yields higher Discr values than the second method. A proportion greater than 0.5 means that the first method demonstrated higher Discr values than the second method on the majority of edges.

Table S3. Differences in the proportion of significant edges between networks derived by MIND-5F and MSN-5F, and between each baseline method and its variants using the Schaefer300 atlas

Dataset	Comparison	Difference (95% CI)
eNKI	MIND-5F vs. MSN-5F	0.091 (0.075, 0.122)
	MIND-5F vs. MIND-CT	-0.146 (-0.169, -0.140)
	MIND-5F vs. MIND-CV	-0.088 (-0.127, -0.072)
	MSN-5F vs. MSN-9F	-0.043 (-0.067, -0.033)
	MSN-5F vs. MSN-5F4S	0.032 (0.011, 0.058)
Cam-CAN	MIND-5F vs. MSN-5F	0.071 (0.051, 0.103)
	MIND-5F vs. MIND-CT	-0.084 (-0.128, -0.067)
	MIND-5F vs. MIND-CV	-0.123 (-0.159, -0.107)
	MSN-5F vs. MSN-9F	-0.004 (-0.024, 0.012)
	MSN-5F vs. MSN-5F4S	0.013 (-0.017, 0.033)

Notes: The *Difference* column indicates the difference in proportion of significant edges between the two networks derived by the methods specified in the *Comparison* column. A positive difference means that the network derived by the first method listed has a higher proportion of significant edges than the network derived by the second method.

Table S4. Differences in mean Cohen’s d measures between networks derived by MIND-5F and MSN-5F, and between each baseline method and its variants in the eNKI dataset

Atlas	Comparison	Difference (95% CI)	Higher-Proportion
DK308	MIND-5F vs. MSN-5F	0.051 (0.042, 0.065)	0.580
	MIND-5F vs. MIND-CT	-0.053 (-0.067, -0.041)	0.413
	MIND-5F vs. MIND-CV	-0.026 (-0.044, -0.014)	0.451
	MSN-5F vs. MSN-9F	-0.012 (-0.019, -0.006)	0.474
	MSN-5F vs. MSN-5F4S	0.007 (-0.002, 0.017)	0.515
Schaefer300	MIND-5F vs. MSN-5F	0.036 (0.027, 0.051)	0.553
	MIND-5F vs. MIND-CT	-0.058 (-0.077, -0.047)	0.403
	MIND-5F vs. MIND-CV	-0.035 (-0.053, -0.024)	0.440
	MSN-5F vs. MSN-9F	-0.014 (-0.023, -0.009)	0.469
	MSN-5F vs. MSN-5F4S	0.013 (0.006, 0.023)	0.524

Notes: The *Difference* column indicates the difference in mean Cohen’s d measures between the two networks derived by the methods specified in the *Comparison* column. A positive difference means that the network derived by the first method listed has a higher mean Cohen’s d than the network derived by the second method. The *Higher-Proportion* column shows the proportion of edges for which the first method yields higher Cohen’s d values than the second method. A proportion greater than 0.5 means that the first method demonstrated higher Cohen’s d values than the second method on the majority of edges.

Table S5. Differences in age prediction accuracy quantified by partial Pearson correlation between MIND-5F and MSN-5F, and between each baseline method and its variants using the Schaefer300 atlas

Dataset	Comparison	Difference (Mean±SD)	T-statistic	P-value
	MIND-5F vs. MSN-5F	0.061±0.017	16.316	<0.001*
	MIND-5F vs. MIND-CT	0.016±0.012	5.854	<0.001*
eNKI	MIND-5F vs. MIND-CV	0.032±0.013	11.010	<0.001*
	MSN-5F vs. MSN-9F	-0.041±0.012	-15.255	<0.001*
	MSN-5F vs. MSN-5F4S	-0.063±0.017	-16.573	<0.001*
	MIND-5F vs. MSN-5F	0.070±0.026	11.891	<0.001*
	MIND-5F vs. MIND-CT	0.018±0.022	3.581	0.002*
Cam-CAN	MIND-5F vs. MIND-CV	0.045±0.021	9.561	<0.001*
	MSN-5F vs. MSN-9F	0.004±0.021	0.872	0.394
	MSN-5F vs. MSN-5F4S	-0.076±0.027	-12.287	<0.001*

Notes: The *Difference* column reports the mean difference in partial Pearson correlation (aggregated across all cross-validation folds) between networks derived by the two methods specified in the *Comparison* column. A positive difference indicates that the network obtained with the first method exhibits higher predictive accuracy than that obtained with the second method. Asterisks in the *P-value* column denote statistical significance at an alpha level of 0.05.

Table S6. Differences in age prediction accuracy quantified by partial Spearman correlation between MIND-5F and MSN-5F, and between each baseline method and its variants across datasets and parcellation atlases.

Dataset	Atlas	Comparison	Difference (Mean±SD)	T-statistic	P-value
eNKI	DK308	MIND-5F vs. MSN-5F	0.034±0.011	13.914	<0.001*
		MIND-5F vs. MIND-CT	0.011±0.010	5.061	<0.001*
		MIND-5F vs. MIND-CV	0.026±0.013	9.289	<0.001*
		MSN-5F vs. MSN-9F	-0.012±0.011	-5.072	<0.001*
		MSN-5F vs. MSN-5F4S	-0.038±0.012	-13.951	<0.001*
	Schaefer300	MIND-5F vs. MSN-5F	0.058±0.014	18.469	<0.001*
		MIND-5F vs. MIND-CT	0.014±0.012	5.213	<0.001*
		MIND-5F vs. MIND-CV	0.030±0.013	10.143	<0.001*
		MSN-5F vs. MSN-9F	-0.039±0.012	-15.152	<0.001*
		MSN-5F vs. MSN-5F4S	-0.060±0.015	-17.582	<0.001*
Cam-CAN	DK308	MIND-5F vs. MSN-5F	0.043±0.018	10.549	<0.001*
		MIND-5F vs. MIND-CT	0.031±0.024	5.723	<0.001*
		MIND-5F vs. MIND-CV	0.038±0.024	7.173	<0.001*
		MSN-5F vs. MSN-9F	-0.006±0.025	-1.044	0.309
		MSN-5F vs. MSN-5F4S	-0.053±0.019	-12.427	<0.001*
	Schaefer300	MIND-5F vs. MSN-5F	0.059±0.026	10.083	<0.001*
		MIND-5F vs. MIND-CT	0.016±0.023	3.029	0.007*
		MIND-5F vs. MIND-CV	0.032±0.018	7.794	<0.001*
		MSN-5F vs. MSN-9F	0.004±0.021	0.792	0.438
		MSN-5F vs. MSN-5F4S	-0.063±0.028	-10.110	<0.001*

Notes: The *Difference* column reports the mean difference in partial Spearman correlation (aggregated across all cross-validation folds) between networks derived by the two methods specified in the *Comparison* column. A positive difference indicates that the network obtained with the first method exhibits higher predictive accuracy than that obtained with the second method. Asterisks in the *P-value* column denote statistical significance at an alpha level of 0.05.

Table S7. Differences in age prediction accuracy quantified by mean absolute error between MIND-5F and MSN-5F, and between each baseline method and its variants across datasets and parcellation atlases.

Dataset	Atlas	Comparison	Difference (Mean±SD)	T-statistic	P-value
eNKI	DK308	MIND-5F vs. MSN-5F	-1.067±0.414	-11.524	<0.001*
		MIND-5F vs. MIND-CT	-0.410±0.331	-5.542	<0.001*
		MIND-5F vs. MIND-CV	-0.994±0.439	-10.124	<0.001*
		MSN-5F vs. MSN-9F	0.287±0.247	5.214	<0.001*
		MSN-5F vs. MSN-5F4S	1.125±0.469	10.724	<0.001*
	Schaefer300	MIND-5F vs. MSN-5F	-1.923±0.392	-21.913	<0.001*
		MIND-5F vs. MIND-CT	-0.495±0.426	-5.202	<0.001*
		MIND-5F vs. MIND-CV	-1.214±0.378	-14.368	<0.001*
		MSN-5F vs. MSN-9F	1.220±0.364	15.004	<0.001*
		MSN-5F vs. MSN-5F4S	1.900±0.337	25.206	<0.001*
Cam-CAN	DK308	MIND-5F vs. MSN-5F	-0.880±0.360	-10.929	<0.001*
		MIND-5F vs. MIND-CT	-0.603±0.405	-6.660	<0.001*
		MIND-5F vs. MIND-CV	-0.815±0.456	-7.982	<0.001*
		MSN-5F vs. MSN-9F	0.117±0.502	1.039	0.312
		MSN-5F vs. MSN-5F4S	1.070±0.361	13.268	<0.001*
	Schaefer300	MIND-5F vs. MSN-5F	-1.185±0.525	-10.103	<0.001*
		MIND-5F vs. MIND-CT	-0.496±0.433	-5.121	<0.001*
		MIND-5F vs. MIND-CV	-0.868±0.397	-9.779	<0.001*
		MSN-5F vs. MSN-9F	-0.180±0.399	-2.013	0.059
		MSN-5F vs. MSN-5F4S	1.040±0.459	10.140	<0.001*

Notes: The *Difference* column reports the mean difference in mean absolute error (aggregated across all cross-validation folds) between networks derived by the two methods specified in the *Comparison* column. A positive difference indicates that the network obtained with the first method exhibits lower predictive accuracy than that obtained with the second method. Asterisks in the *P-value* column denote statistical significance at an alpha level of 0.05.

Table S8. Differences in sex-stratified age prediction accuracy between MIND-5F and MSN-5F, and between each baseline method and its variants in the eNKI dataset using the DK308 atlas.

Metric	Comparison	Sex	Difference (Mean±SD)	T-statistic	P-value
PPC	MIND-5F vs. MSN-5F	F	0.035±0.013	11.981	<0.001*
		M	0.033±0.020	7.474	<0.001*
	MIND-5F vs. MIND-CT	F	0.013±0.012	5.036	<0.001*
		M	0.010±0.012	3.582	0.002*
	MIND-5F vs. MIND-CV	F	0.032±0.012	12.149	<0.001*
		M	0.025±0.017	6.569	<0.001*
	MSN-5F vs. MSN-9F	F	-0.013±0.012	-4.913	<0.001*
		M	-0.010±0.014	-2.949	0.008*
	MSN-5F vs. MSN-5F4S	F	-0.043±0.016	-11.720	<0.001*
		M	-0.033±0.018	-8.179	<0.001*
PSC	MIND-5F vs. MSN-5F	F	0.038 ±0.014	12.033	<0.001*
		M	0.044±0.022	8.873	<0.001*
	MIND-5F vs. MIND-CT	F	0.013±0.014	4.071	<0.001*
		M	0.015±0.015	4.424	<0.001*
	MIND-5F vs. MIND-CV	F	0.025±0.013	8.553	<0.001*
		M	0.030±0.021	6.292	<0.001*
	MSN-5F vs. MSN-9F	F	-0.016±0.016	-4.318	<0.001*
		M	-0.014±0.016	-3.931	<0.001*
	MSN-5F vs. MSN-5F4S	F	-0.047±0.018	-11.693	<0.001*
		M	-0.043±0.019	-10.088	<0.001*
MAE	MIND-5F vs. MSN-5F	F	-1.065±0.386	-12.326	<0.001*
		M	-1.074±0.671	-7.162	<0.001*
	MIND-5F vs. MIND-CT	F	-0.452±0.459	-4.399	<0.001*
		M	-0.314±0.429	-3.279	0.004*
	MIND-5F vs. MIND-CV	F	-1.088±0.427	-11.401	<0.001*
		M	-0.858±0.594	-6.456	<0.001*
	MSN-5F vs. MSN-9F	F	0.275±0.321	3.828	0.001*
		M	0.303±0.467	2.906	0.009*

	MIND-5F vs. MSN-5F4S	F	1.181±0.573	9.213	<0.001*
		M	0.936±0.631	6.633	<0.001*

Notes: The *Difference* column reports the mean difference in prediction accuracy metric (aggregated across all cross-validation folds) between networks derived by the two methods specified in the *Comparison* column. For partial Pearson correlation (PPC) and partial Spearman correlation (PSC), a positive difference indicates that the network obtained with the first method exhibits higher predictive accuracy than that obtained with the second method. For mean absolute error (MAE), a positive difference indicates that the first method exhibits lower predictive accuracy than the second method. Asterisks in the *P-value* column denote statistical significance at an alpha level of 0.05.

Table S9. Differences in sex-stratified age prediction accuracy between MIND-5F and MSN-5F, and between each baseline method and its variants in the Cam-CAN dataset using the DK308 atlas.

Metric	Comparison	Sex	Difference (Mean±SD)	T-statistic	P-value
PPC	MIND-5F vs. MSN-5F	F	0.068±0.039	7.848	<0.001*
		M	0.036±0.038	4.288	<0.001*
	MIND-5F vs. MIND-CT	F	0.017±0.031	2.374	0.028*
		M	0.044±0.025	7.922	<0.001*
	MIND-5F vs. MIND-CV	F	0.041±0.050	3.653	0.002*
		M	0.069±0.049	6.304	<0.001*
	MSN-5F vs. MSN-9F	F	-0.006±0.040	-0.661	0.517
		M	-0.005±0.040	-0.580	0.568
	MSN-5F vs. MSN-5F4S	F	-0.062±0.025	-11.270	<0.001*
		M	-0.063±0.048	-5.859	<0.001*
PSC	MIND-5F vs. MSN-5F	F	0.065±0.046	6.336	<0.001*
		M	0.025±0.032	3.528	0.002*
	MIND-5F vs. MIND-CT	F	0.023±0.037	2.753	0.013*
		M	0.045±0.032	6.371	<0.001*
	MIND-5F vs. MIND-CV	F	0.038±0.050	3.412	0.003*
		M	0.058±0.045	5.662	<0.001*
	MSN-5F vs. MSN-9F	F	-0.003±0.042	-0.289	0.775
		M	-0.009±0.032	-1.227	0.235
	MSN-5F vs. MSN-5F4S	F	-0.056±0.039	-6.448	<0.001*

		M	-0.053±0.045	-5.220	<0.001*
MAE	MIND-5F vs. MSN-5F	F	-1.051±0.732	-6.421	<0.001*
		M	-0.499±0.548	-4.069	<0.001*
	MIND-5F vs. MIND-CT	F	-0.270±0.628	-1.921	0.070
		M	-0.807±0.645	-5.593	<0.001*
	MIND-5F vs. MIND-CV	F	-0.589±0.806	-3.266	0.004*
		M	-0.948±0.731	-5.803	<0.001*
	MSN-5F vs. MSN-9F	F	0.155±0.505	1.371	0.186
		M	0.057±0.758	0.335	0.741
	MIND-5F vs. MSN-5F4S	F	0.986±0.499	8.837	<0.001*
		M	1.075±0.661	7.272	<0.001*

Notes: The *Difference* column reports the mean difference in prediction accuracy metric (aggregated across all cross-validation folds) between networks derived by the two methods specified in the *Comparison* column. For partial Pearson correlation (PPC) and partial Spearman correlation (PSC), a positive difference indicates that the network obtained with the first method exhibits higher predictive accuracy than that obtained with the second method. For mean absolute error (MAE), a positive difference indicates that the first method exhibits lower prediction accuracy than the second method. Asterisks in the *P-value* column denote statistical significance at an alpha level of 0.05.

Table S10. Differences in sex-stratified age prediction accuracy between MIND-5F and MSN-5F, and between each baseline method and its variants in the eNKI dataset using the Schaefer300 atlas.

Metric	Comparison	Sex	Difference (Mean±SD)	T-statistic	P-value
PPC	MIND-5F vs. MSN-5F	F	0.057±0.021	11.976	<0.001*
		M	0.069±0.025	12.234	<0.001*
	MIND-5F vs. MIND-CT	F	0.015±0.017	4.102	<0.001*
		M	0.016±0.012	6.043	<0.001*
	MIND-5F vs. MIND-CV	F	0.032±0.018	8.147	<0.001*
		M	0.032±0.015	9.664	<0.001*
	MSN-5F vs. MSN-9F	F	-0.042±0.014	-13.423	<0.001*
		M	-0.039±0.022	-7.997	<0.001*
	MSN-5F vs. MSN-5F4S	F	-0.061±0.022	-12.600	<0.001*
		M	-0.066±0.026	-11.577	<0.001*

PSC	MIND-5F vs. MSN-5F	F	0.058 ±0.021	12.236	<0.001*
		M	0.065±0.025	11.495	<0.001*
	MIND-5F vs. MIND-CT	F	0.017±0.018	4.104	<0.001*
		M	0.017±0.015	4.807	<0.001*
	MIND-5F vs. MIND-CV	F	0.028±0.019	6.510	<0.001*
		M	0.036±0.018	8.958	<0.001*
	MSN-5F vs. MSN-9F	F	-0.041±0.016	-11.781	<0.001*
		M	-0.039±0.024	-7.242	<0.001*
	MSN-5F vs. MSN-5F4S	F	-0.066±0.024	-12.188	<0.001*
		M	-0.064±0.028	-10.182	<0.001*
MAE	MIND-5F vs. MSN-5F	F	-1.771±0.464	-17.065	<0.001*
		M	-2.054±0.757	-12.132	<0.001*
	MIND-5F vs. MIND-CT	F	-0.430±0.523	-3.677	0.002*
		M	-0.562±0.491	-5.123	<0.001*
	MIND-5F vs. MIND-CV	F	-1.133±0.478	-10.592	<0.001*
		M	-1.413±0.472	-13.395	<0.001*
	MSN-5F vs. MSN-9F	F	1.288±0.457	12.601	<0.001*
		M	0.985±0.572	7.695	<0.001*
	MIND-5F vs. MSN-5F4S	F	1.766±0.402	19.645	<0.001*
		M	1.810±0.594	13.627	<0.001*

Notes: The *Difference* column reports the mean difference in prediction accuracy metric (aggregated across all cross-validation folds) between networks derived by the two methods specified in the *Comparison* column. For partial Pearson correlation (PPC) and partial Spearman correlation (PSC), a positive difference indicates that the network obtained with the first method exhibits higher predictive accuracy than that obtained with the second method. For mean absolute error (MAE), a positive difference indicates that the first method exhibits lower predictive accuracy than the second method. Asterisks in the *P-value* column denote statistical significance at an alpha level of 0.05.

Table S11. Differences in sex-stratified age prediction accuracy between MIND-5F and MSN-5F, and between each baseline method and its variants in the Cam-CAN dataset using the Schaefer300 atlas.

Metric	Comparison	Sex	Difference (Mean±SD)	T-statistic	P-value

PPC	MIND-5F vs. MSN-5F	F	0.072±0.034	9.475	<0.001*
		M	0.063±0.047	6.056	<0.001*
	MIND-5F vs. MIND-CT	F	0.008±0.028	1.213	0.240
		M	0.024±0.030	3.591	0.002*
	MIND-5F vs. MIND-CV	F	0.031±0.035	4.028	<0.001*
		M	0.056±0.030	8.290	<0.001*
	MSN-5F vs. MSN-9F	F	0.003±0.026	0.479	0.638
		M	0.010±0.037	1.205	0.243
	MSN-5F vs. MSN-5F4S	F	-0.070±0.034	-9.029	<0.001*
		M	-0.078±0.045	-7.692	<0.001*
PSC	MIND-5F vs. MSN-5F	F	0.059 ±0.036	7.228	<0.001*
		M	0.068±0.047	6.422	<0.001*
	MIND-5F vs. MIND-CT	F	0.007±0.026	1.263	0.222
		M	0.023±0.034	3.110	0.006*
	MIND-5F vs. MIND-CV	F	0.024±0.035	3.054	0.007*
		M	0.049±0.034	6.424	<0.001*
	MSN-5F vs. MSN-9F	F	0.016±0.030	2.353	0.030*
		M	-0.002±0.035	-0.300	0.768
	MSN-5F vs. MSN-5F4S	F	-0.052±0.039	-5.939	<0.001*
		M	-0.081±0.051	-7.113	<0.001*
MAE	MIND-5F vs. MSN-5F	F	-1.078±0.602	-8.011	<0.001*
		M	-1.125±0.898	-5.600	<0.001*
	MIND-5F vs. MIND-CT	F	-0.182±0.535	-1.523	0.144
		M	-0.802±0.735	-4.883	<0.001*
	MIND-5F vs. MIND-CV	F	-0.295±0.606	-2.179	0.042*
		M	-1.445±0.580	-11.135	<0.001*
	MSN-5F vs. MSN-9F	F	-0.140±0.485	-1.287	0.213
		M	-0.359±0.638	-2.516	0.021*
	MIND-5F vs. MSN-5F4S	F	0.855±0.534	7.164	<0.001*
		M	1.067±0.834	5.719	<0.001*

Notes: The *Difference* column reports the mean difference in prediction accuracy metric (aggregated across all cross-validation folds) between networks derived by the two methods

specified in the *Comparison* column. For partial Pearson correlation (PPC) and partial Spearman correlation (PSC), a positive difference indicates that the network obtained with the first method exhibits higher predictive accuracy than that obtained with the second method. For mean absolute error (MAE), a positive difference indicates that the first method exhibits lower predictive accuracy than the second method. Asterisks in the *P-value* column denote statistical significance at an alpha level of 0.05.

Table S12. Differences in age prediction accuracy between MIND-5F and MSN-5F4S across datasets and parcellation atlases.

Dataset	Atlas	Metric	Difference (Mean±SD)	T-statistic	P-value
eNKI	DK308	PPC	-0.004±0.007	-2.651	0.016*
		PSC	-0.004±0.007	-2.926	0.009*
		MAE	0.058±0.322	0.805	0.431
	Schaefer300	PPC	-0.001±0.010	-0.614	0.547
		PSC	-0.002±0.009	-0.906	0.376
		MAE	-0.023±0.282	-0.366	0.718
Cam-CAN	DK308	PPC	-0.011±0.018	-2.722	0.014*
		PSC	-0.010±0.016	-2.830	0.011*
		MAE	0.190±0.401	2.122	0.047*
	Schaefer300	PPC	-0.005±0.015	-1.522	0.145
		PSC	-0.003±0.014	-1.078	0.295
		MAE	-0.145±0.328	-1.979	0.063

Notes: The *Difference* column reports the mean difference in prediction accuracy metric (aggregated across all cross-validation folds) between networks derived by MIND-5F and MSN-5F4S. For partial Pearson correlation (PPC) and partial Spearman correlation (PSC), a positive difference indicates higher predictive accuracy with MIND-5F than with MSN-5F4S. For mean absolute error (MAE), a positive difference indicates lower predictive accuracy with MIND-5F. Asterisks in the *P-value* column denote statistical significance at an alpha level of 0.05.

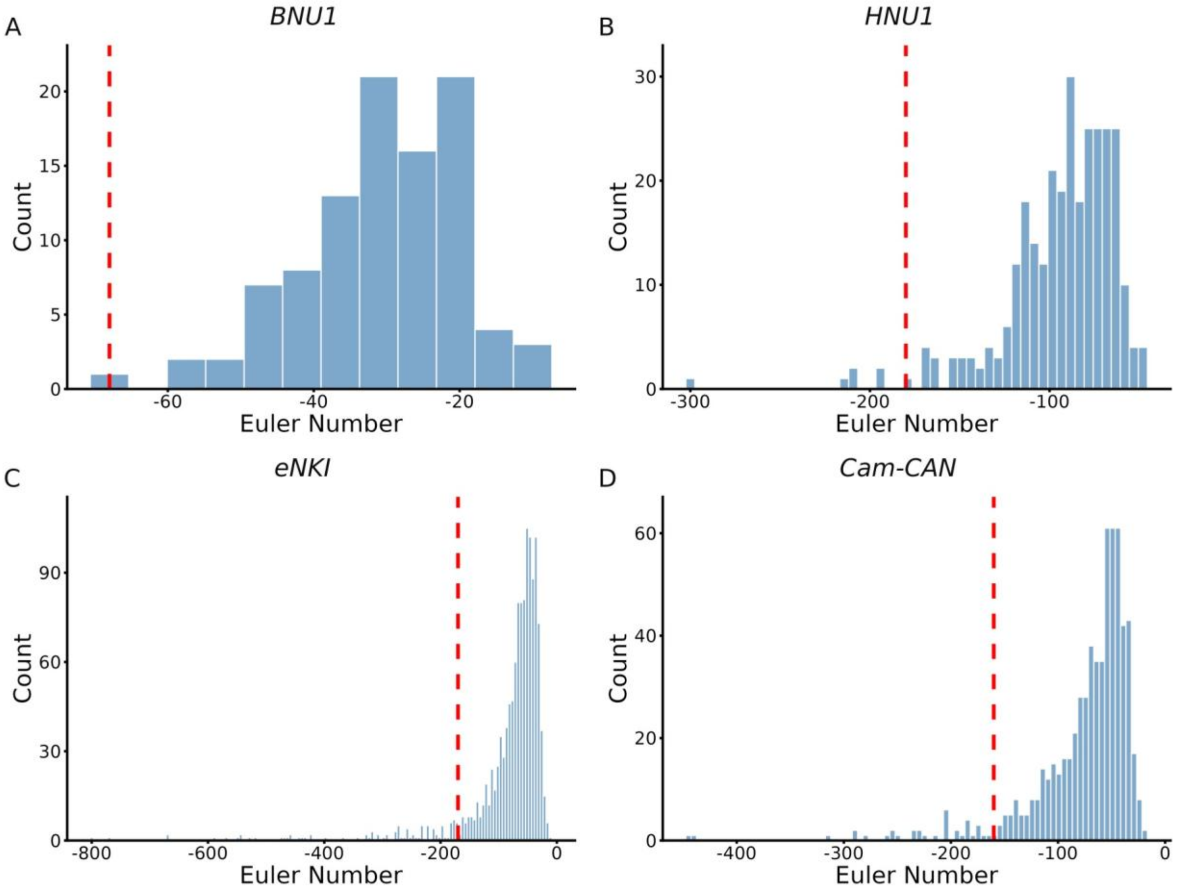

Figure S1. Histogram of Euler numbers for all participants and sessions. The vertical red dashed line marks the largest Euler number with a modified Z-score below -3.5.

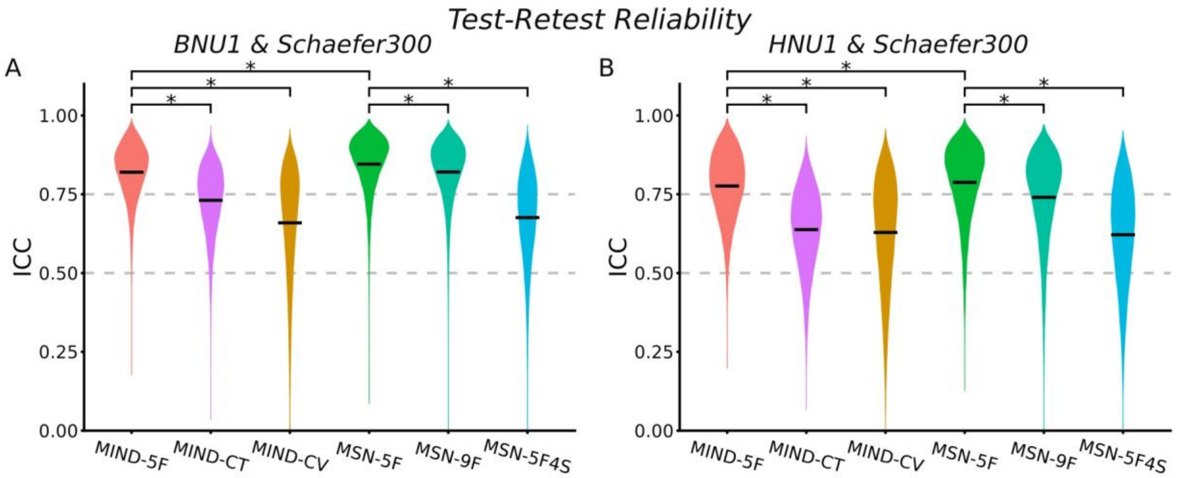

Figure S2. Violin plots of edge-wise intraclass correlation coefficients (ICCs) for networks derived by MIND-5F, MSN-5F, and their variants using the Schaefer300 atlas in (A) the BNU1 dataset and (B) the HNU1 dataset. Statistical differences in mean ICCs between networks derived by MIND-5F and MSN-5F, as well as between networks derived by MIND-5F/MSN-5F and their respective variants, were assessed via bootstrapping. The horizontal black bar within each violin represents the mean ICC for the corresponding network. Asterisks indicate statistical significance at an alpha level of 0.05.

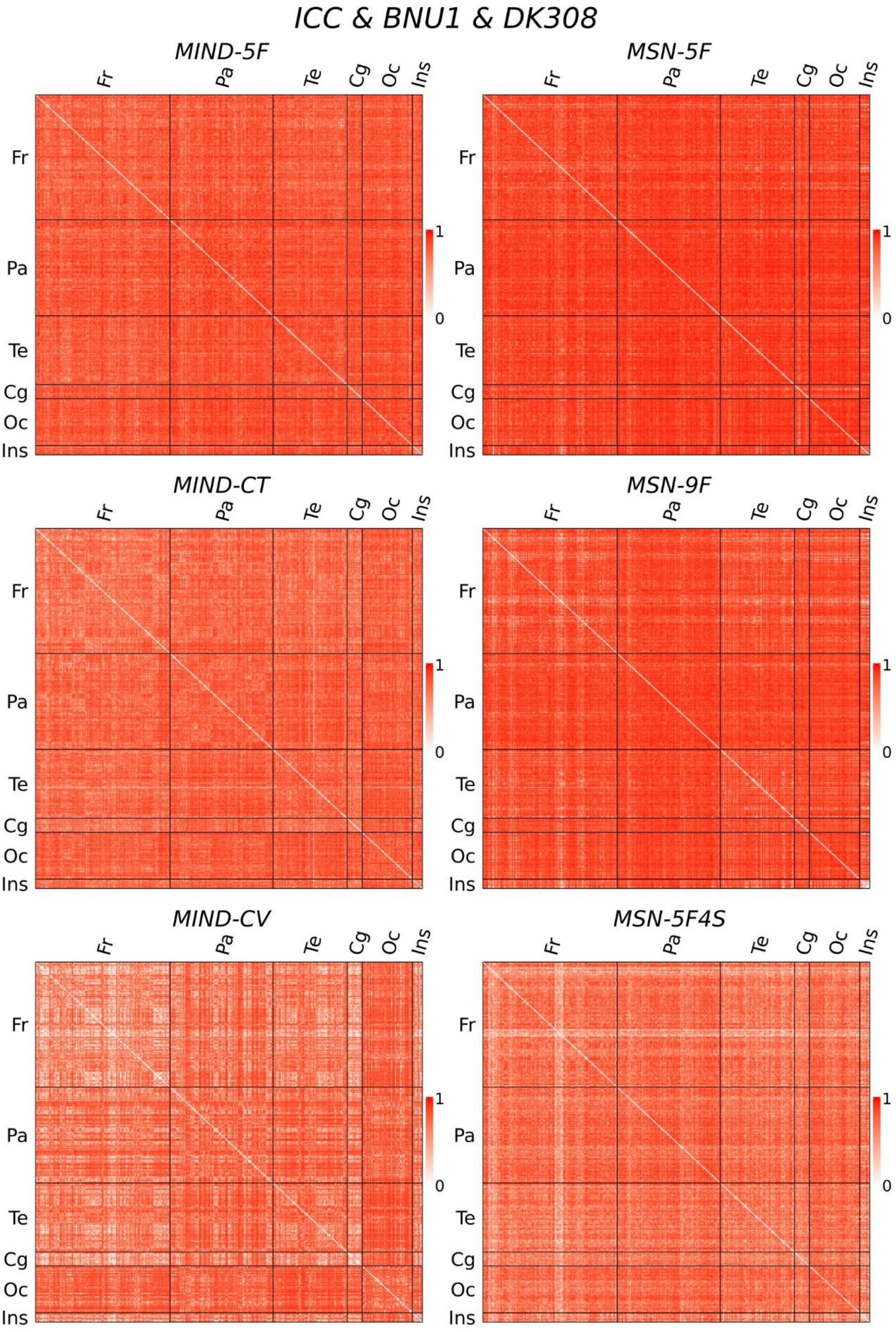

Figure S3. ICC matrices for networks derived by MIND-5F, MSN-5F, and their variants using the DK308 atlas in the BNU1 dataset. Rows and columns are clustered according to the lobar location of each brain region. Abbreviations: Fr, frontal lobe; Pa, parietal lobe; Te, temporal lobe; Cg, cingulate cortex; Oc, occipital lobe; Ins, insula.

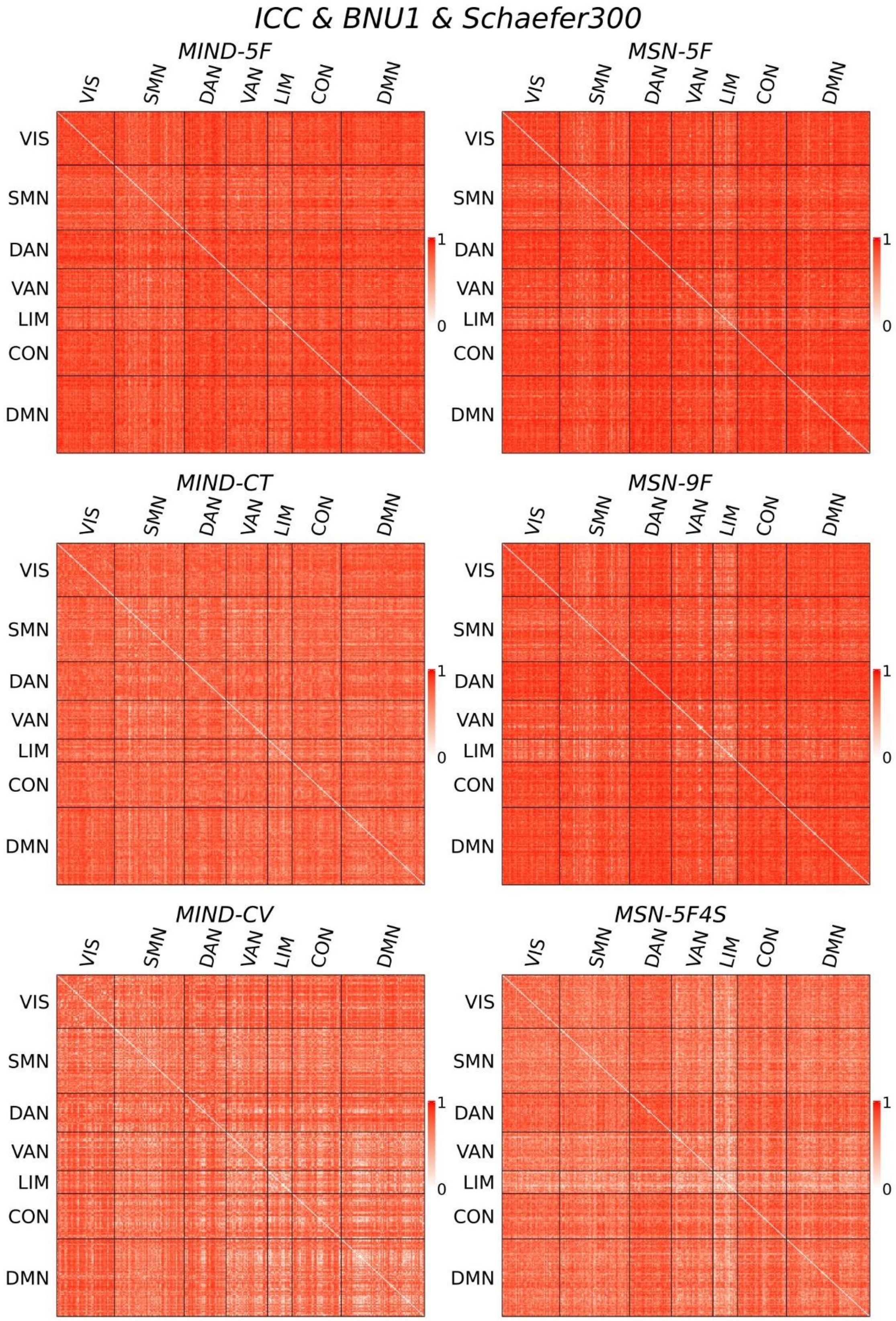

Figure S4. ICC matrices for networks derived by MIND-5F, MSN-5F, and their variants using the Schaefer300 atlas in the BNU1 dataset. Rows and columns are clustered based on the network assignment of each brain region. Abbreviations: VIS, visual network; SMN, somatomotor network; DAN, dorsal attention network; VAN, ventral attention network; LIM, limbic network; CON, frontoparietal control network; DMN, default mode network.

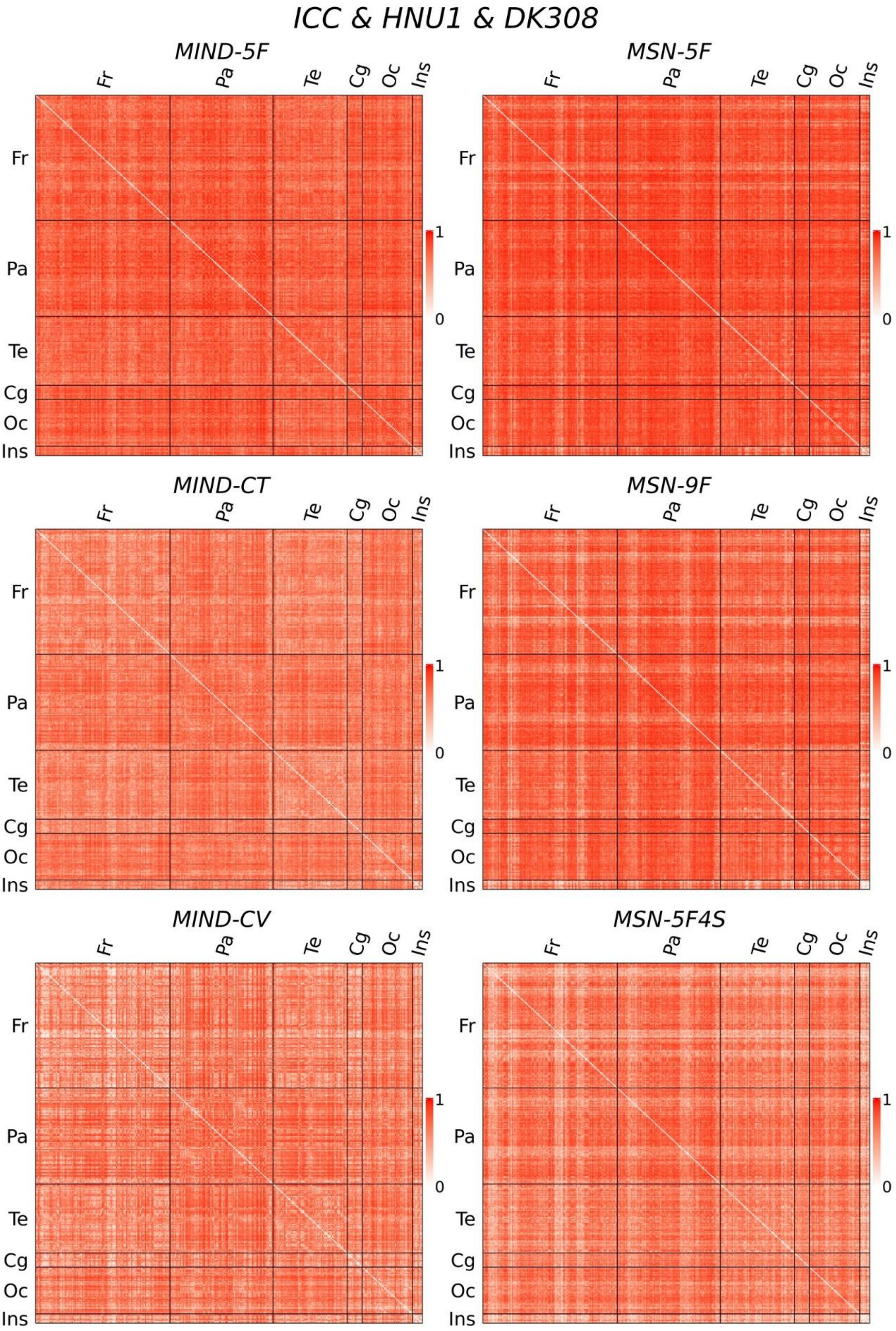

Figure S5. ICC matrices for networks derived by MIND-5F, MSN-5F, and their variants using the DK308 atlas in the HNU1 dataset. Rows and columns are clustered according to the lobar location of each brain region. Abbreviations: Fr, frontal lobe; Pa, parietal lobe; Te, temporal lobe; Cg, cingulate cortex; Oc, occipital lobe; Ins, insula.

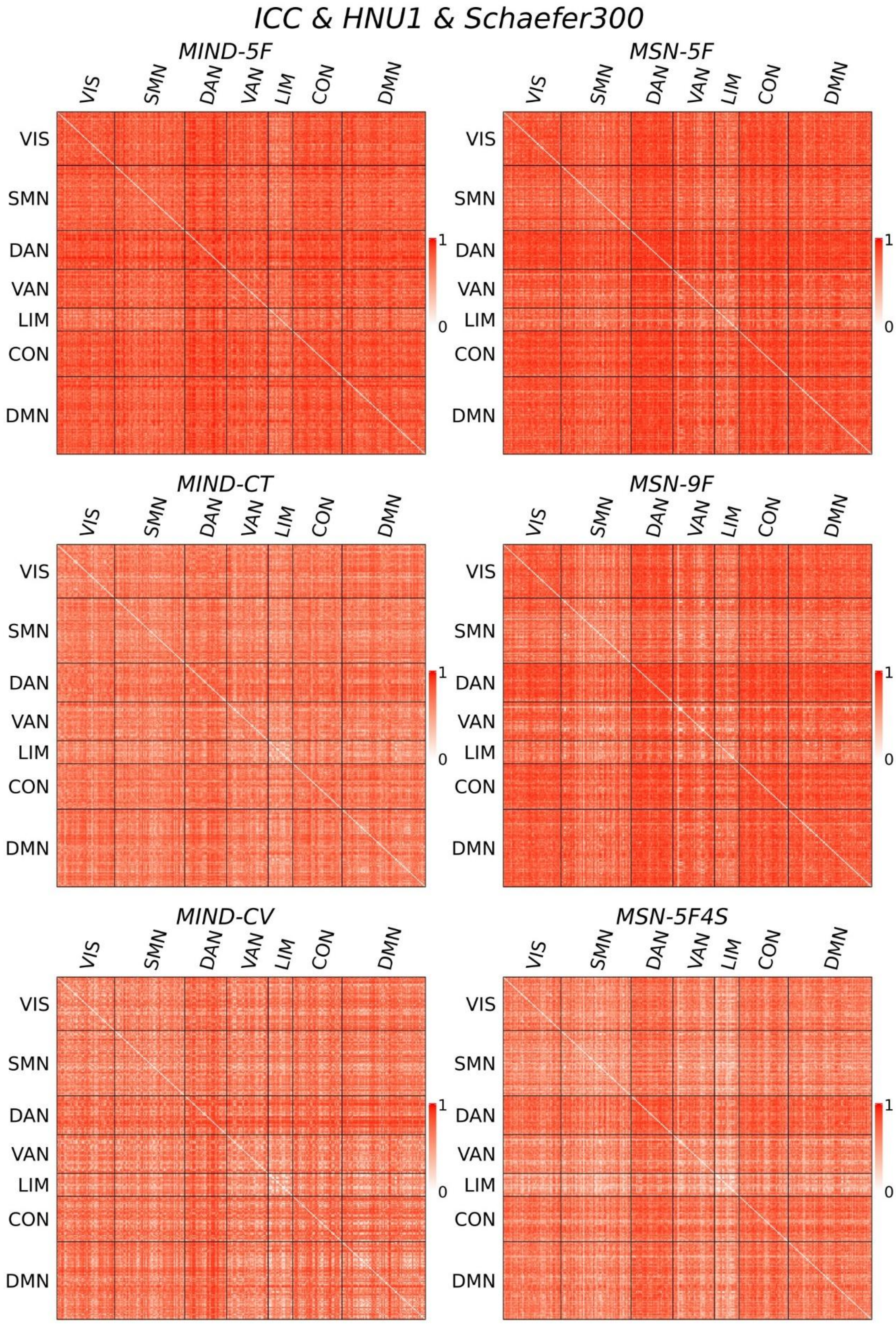

Figure S6. ICC matrices for networks derived by MIND-5F, MSN-5F, and their variants using the Schaefer300 atlas in the HNU1 dataset. Rows and columns are clustered based on the network assignment of each brain region. Abbreviations: VIS, visual network; SMN, somatomotor network; DAN, dorsal attention network; VAN, ventral attention network; LIM, limbic network; CON, frontoparietal control network; DMN, default mode network.

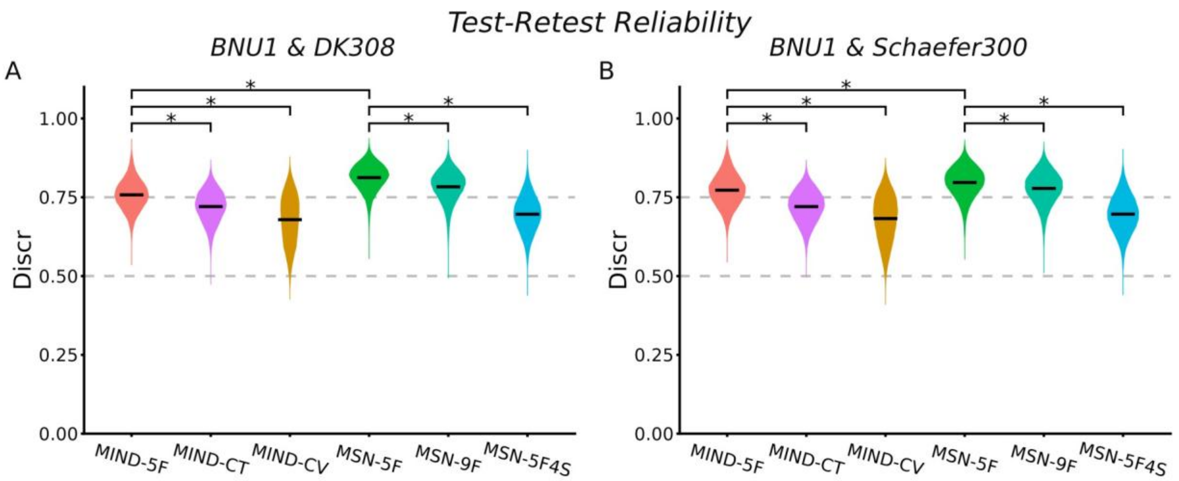

Figure S7. Violin plots of edge-wise Discr statistics for networks derived by MIND-5F, MSN-5F, and their variants using (A) the DK308 atlas and (B) the Schaefer300 atlas in the BNU1 dataset. Statistical differences in mean Discr values between networks derived by MIND-5F and MSN-5F, as well as between networks derived by MIND-5F/MSN-5F and their respective variants, were assessed via bootstrapping. The horizontal black bar within each violin represents the mean Discr for the corresponding network. Asterisks indicate statistical significance at an alpha level of 0.05.

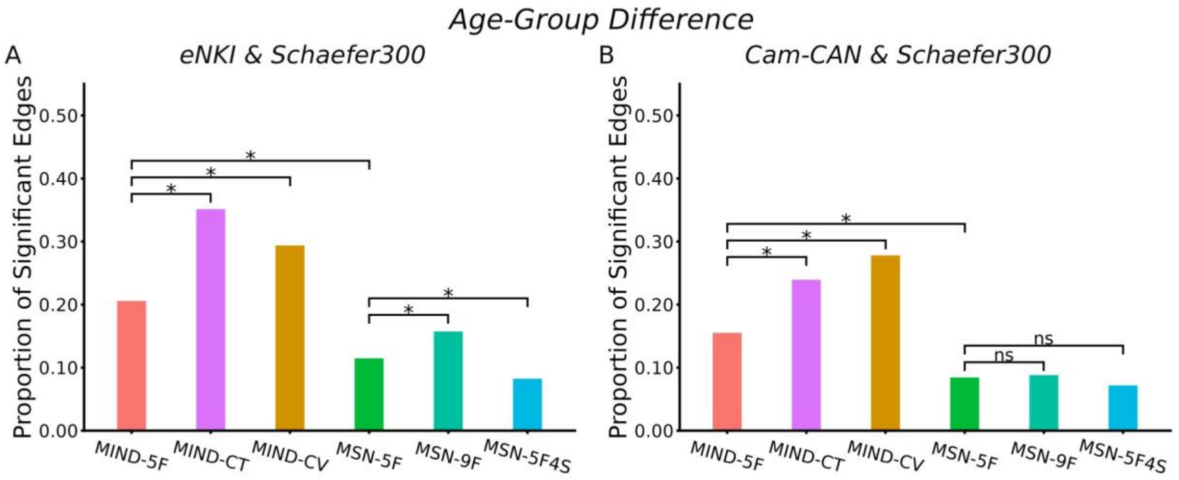

Figure S8. Proportions of significant edges comparing younger and older age groups for networks derived by MIND-5F, MSN-5F, and their variants using the Schaefer300 atlas in (A) the eNKI dataset and (B) the Cam-CAN dataset. Statistical differences in these proportions between networks derived by MIND-5F and MSN-5F, as well as between networks derived by MIND-5F/MSN-5F and their respective variants, were assessed via bootstrapping. Asterisks indicate statistical significance at an alpha level of 0.05, while “ns” denotes non-significance.

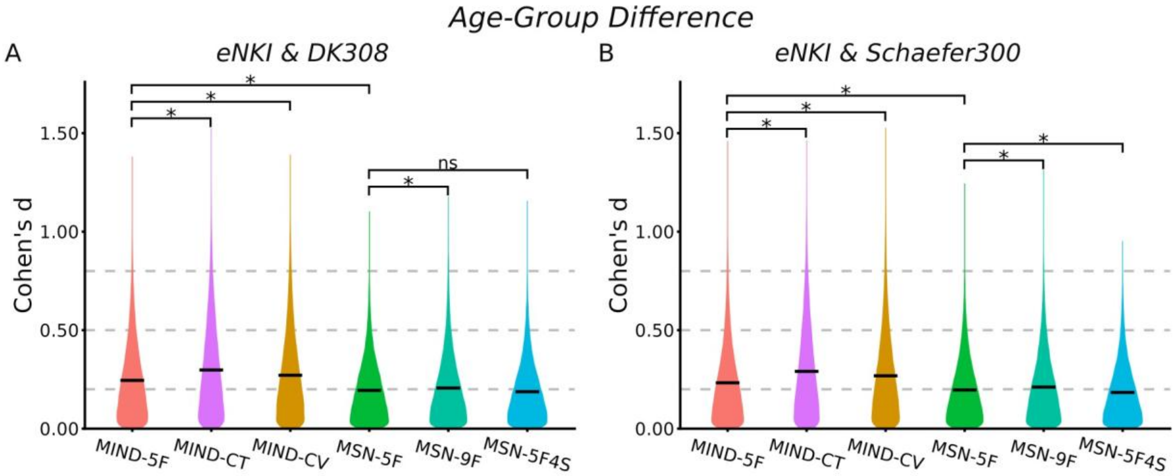

Figure S9. Violin plots of edge-wise Cohen's d measures for networks derived by MIND-5F, MSN-5F, and their variants using (A) the DK308 atlas and (B) the Schaefer300 atlas in the eNKI dataset. Statistical differences in mean Cohen's d values between networks derived by MIND-5F and MSN-5F, as well as between networks derived by MIND-5F/MSN-5F and their respective variants, were assessed via bootstrapping. The horizontal black bar within each violin represents the mean Cohen's d for the corresponding network. Asterisks indicate statistical significance at an alpha level of 0.05.

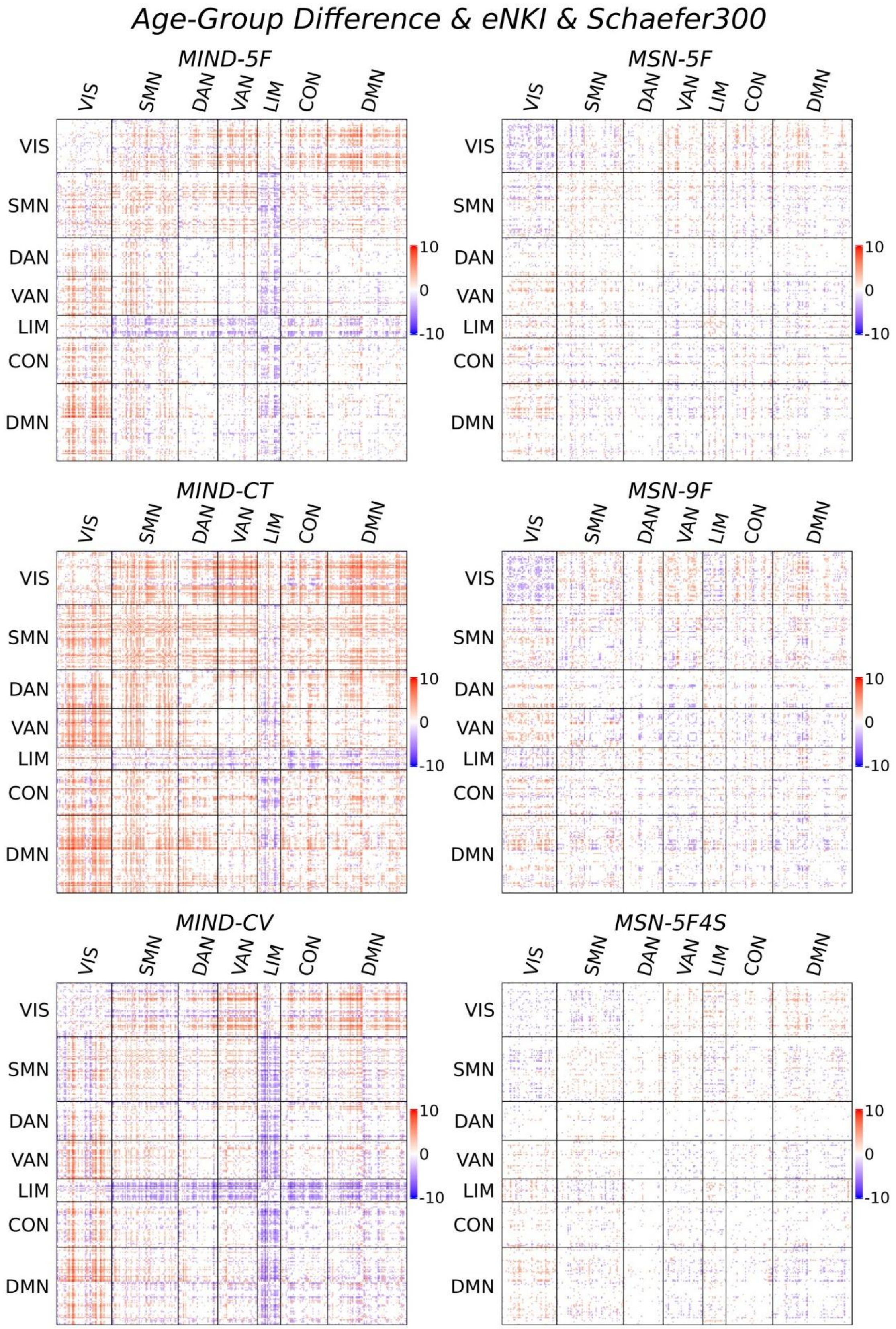

Figure S10. T-statistic matrices comparing younger and older age groups for networks derived by MIND-5F, MSN-5F, and their variants using the Schaefer300 atlas in the eNKI dataset. Positive t-statistic values indicate higher edge weights in the older group compared to the younger group; negative values indicate the opposite. Non-significant matrix entries are set to zero. Rows and columns are clustered based on the network assignment of each brain region. Abbreviations: VIS,

visual network; SMN, somatomotor network; DAN, dorsal attention network; VAN, ventral attention network; LIM, limbic network; CON, frontoparietal control network; DMN, default mode network.

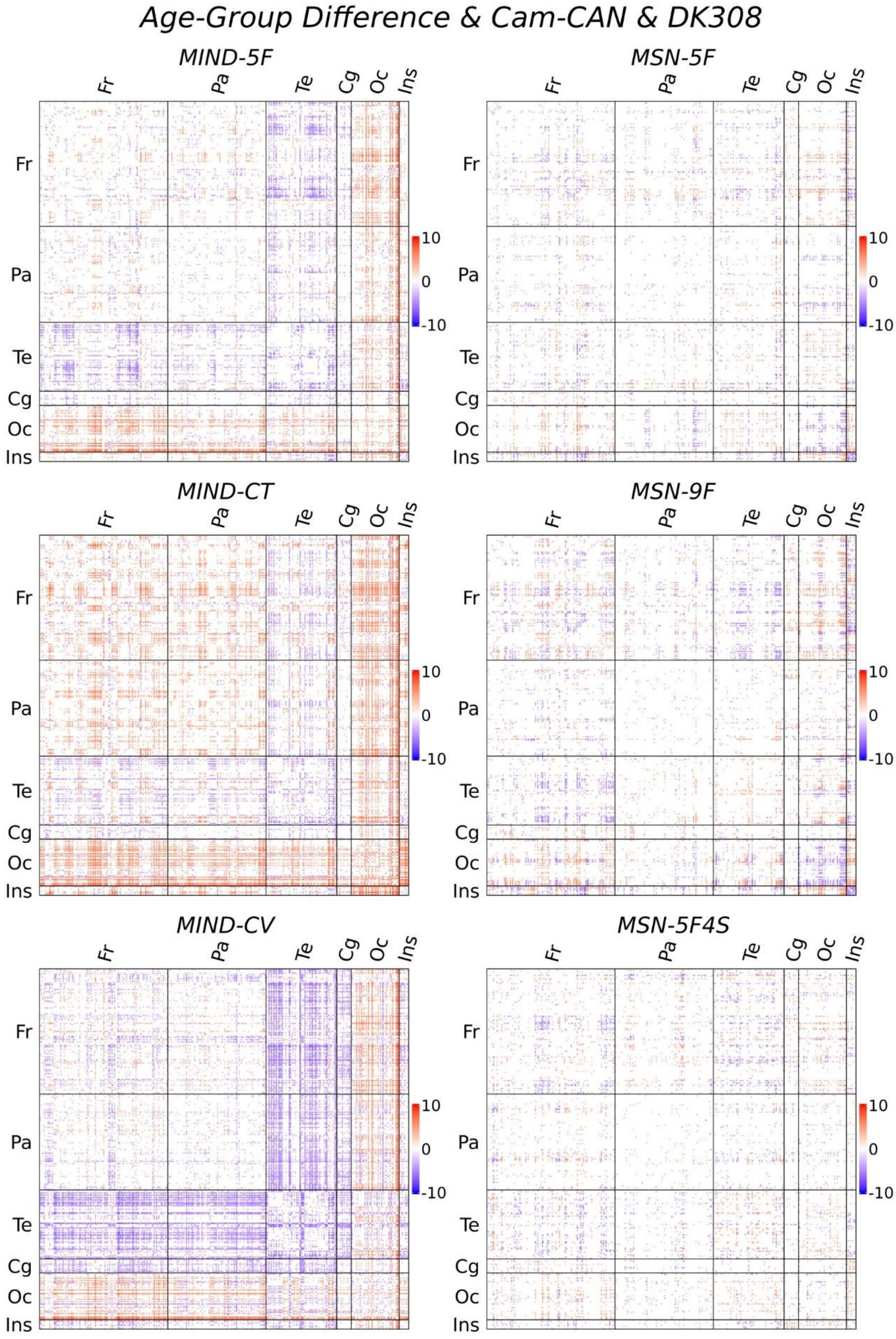

Figure S11. T-statistic matrices comparing younger and older age groups for networks derived by MIND-5F, MSN-5F, and their variants using the DK308 atlas in the Cam-CAN dataset. Positive t-statistic values indicate higher edge weights in the older group compared to the younger group; negative values indicate the opposite. Non-significant matrix entries are set to zero. Rows and columns are clustered according to the lobar location of each brain region. Abbreviations: Fr, frontal lobe; Pa, parietal lobe; Te, temporal lobe; Cg, cingulate cortex; Oc, occipital lobe; Ins, insula.

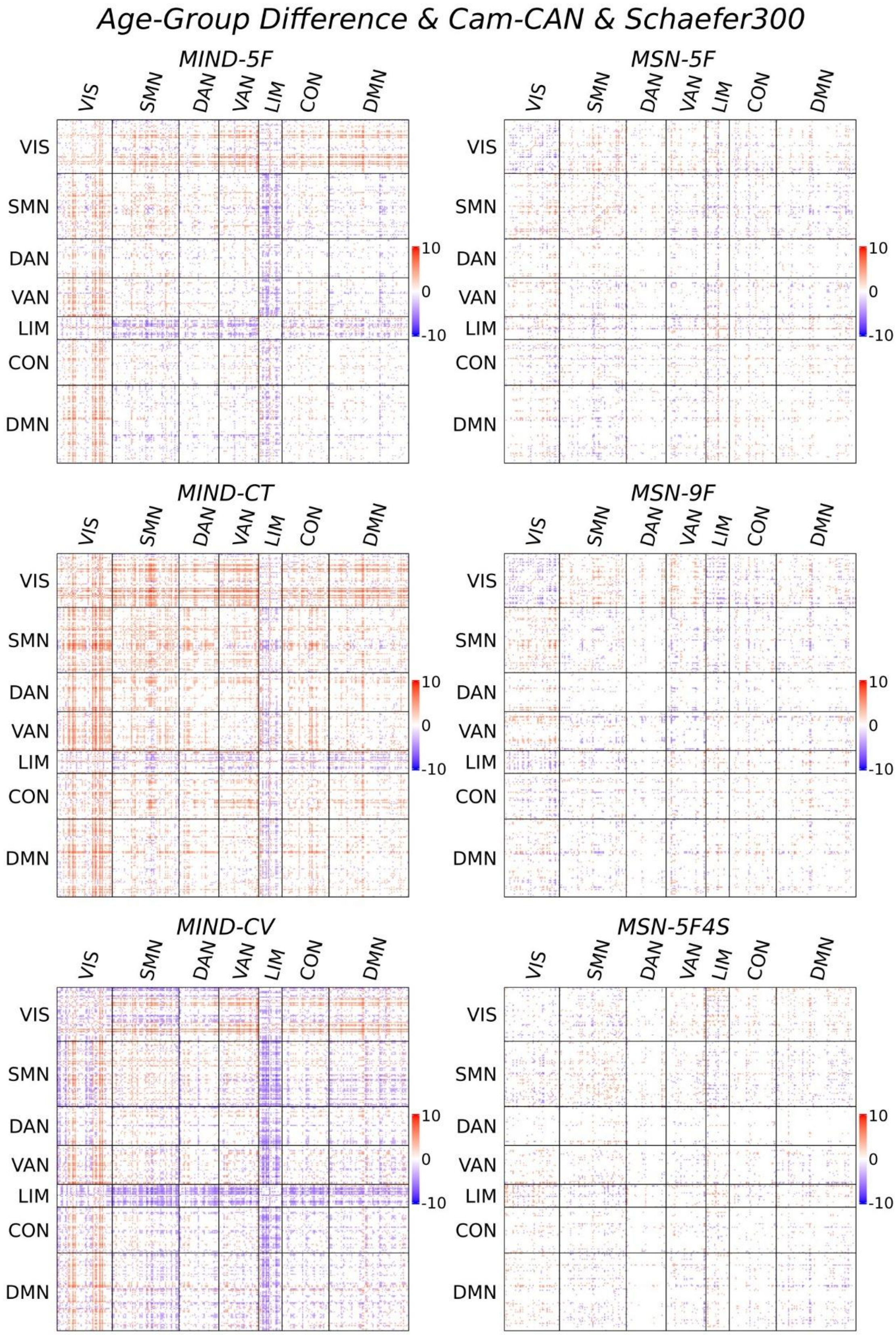

Figure S12. T-statistic matrices comparing younger and older age groups for networks derived by MIND-5F, MSN-5F, and their variants using the Schaefer300 atlas in the Cam-CAN dataset. Positive t-statistic values indicate higher edge weights in the older group compared to the younger group; negative values indicate the opposite. Non-significant matrix entries are set to zero. Rows and columns are clustered based on the network assignment of each brain region. Abbreviations:

VIS, visual network; SMN, somatomotor network; DAN, dorsal attention network; VAN, ventral attention network; LIM, limbic network; CON, frontoparietal control network; DMN, default mode network.

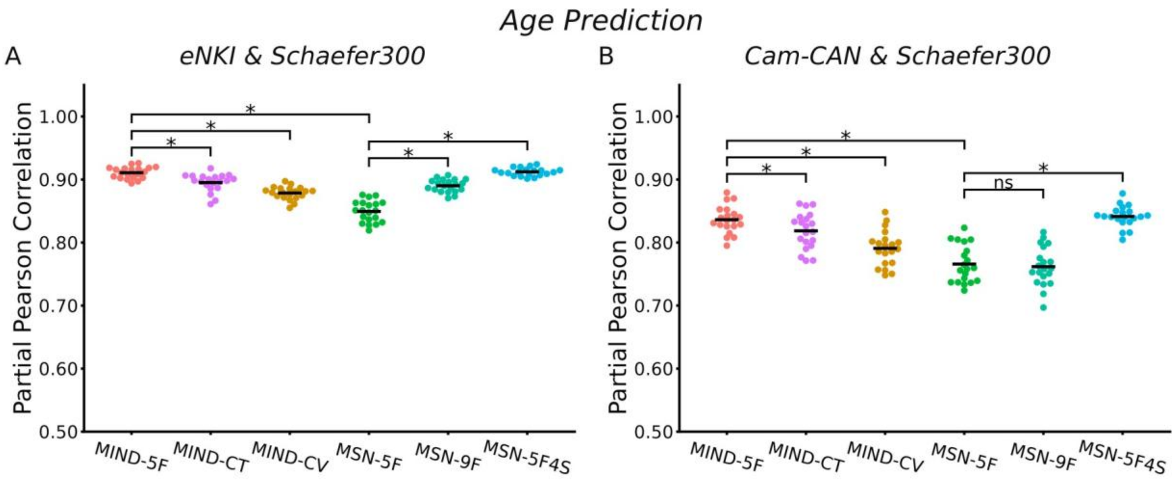

Figure S13. Age prediction performance of models trained on networks derived by MIND-5F, MSN-5F, and their variants using the Schaefer300 atlas in (A) the eNKI dataset and (B) the Cam-CAN dataset. A 5-fold cross-validation procedure was repeated four times to yield 20 accuracy estimates, each represented by a point in the scatter plot. In each fold, prediction accuracy was quantified by the partial Pearson correlation between predicted and true age, with sex, TIV, and Euler number as covariates. Statistical differences in prediction accuracy between networks derived by MIND-5F and MSN-5F, as well as between networks derived by MIND-5F/MSN-5F and their respective variants, were assessed using paired t-tests. Asterisks indicate statistical significance at an alpha level of 0.05, while "ns" denotes non-significance.

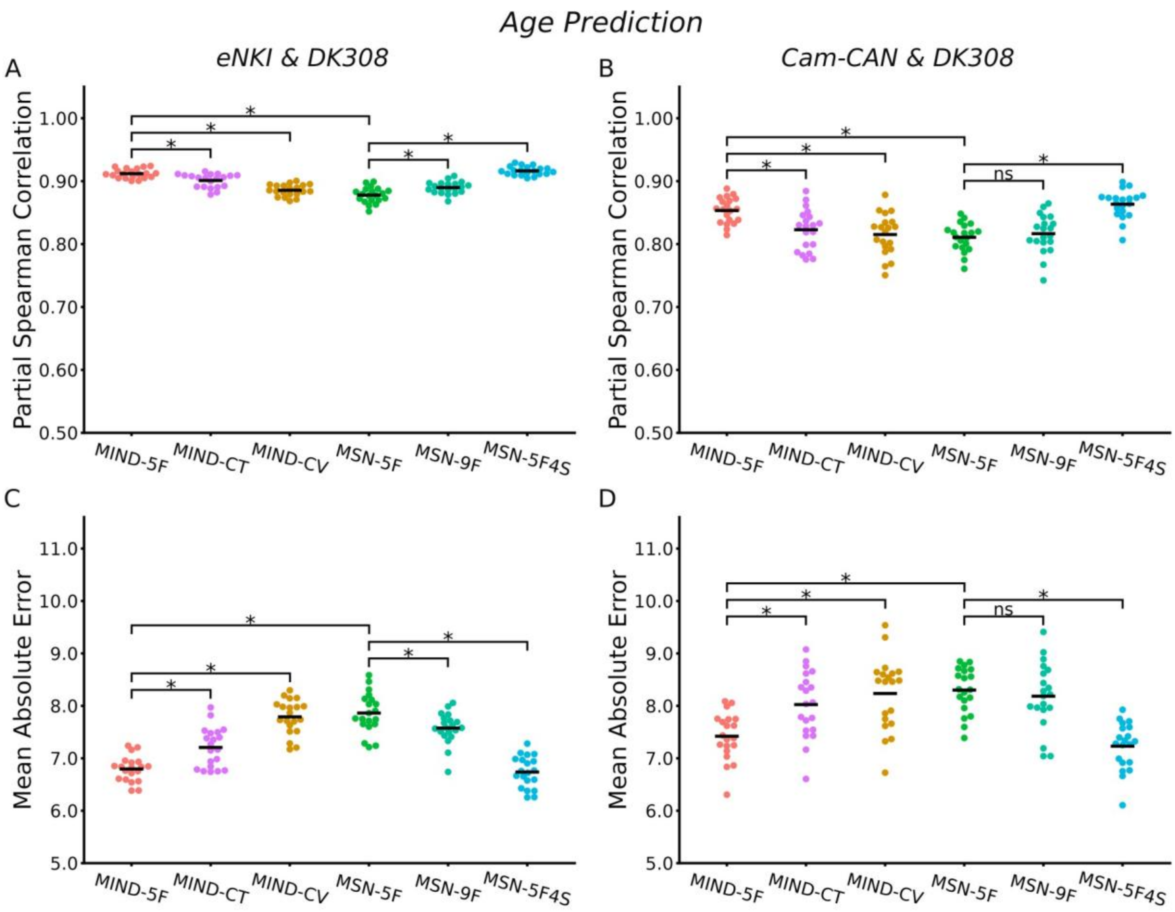

Figure S14. Age prediction performance of models trained on networks derived by MIND-5F, MSN-5F, and their variants using the DK308 atlas in (A, C) the eNKI dataset and (B, D) the Cam-CAN dataset. A 5-fold cross-validation procedure was repeated four times to yield 20 accuracy estimates, each represented by a point in the scatter plot. In each fold, prediction accuracy was quantified by (A, B) the partial Spearman correlation and (C, D) the mean absolute error between predicted and true age. Statistical differences in prediction accuracy between networks derived by MIND-5F and MSN-5F, as well as between networks derived by MIND-5F/MSN-5F and their respective variants, were assessed using paired t-tests. Asterisks indicate statistical significance at an alpha level of 0.05, while “ns” denotes non-significance.

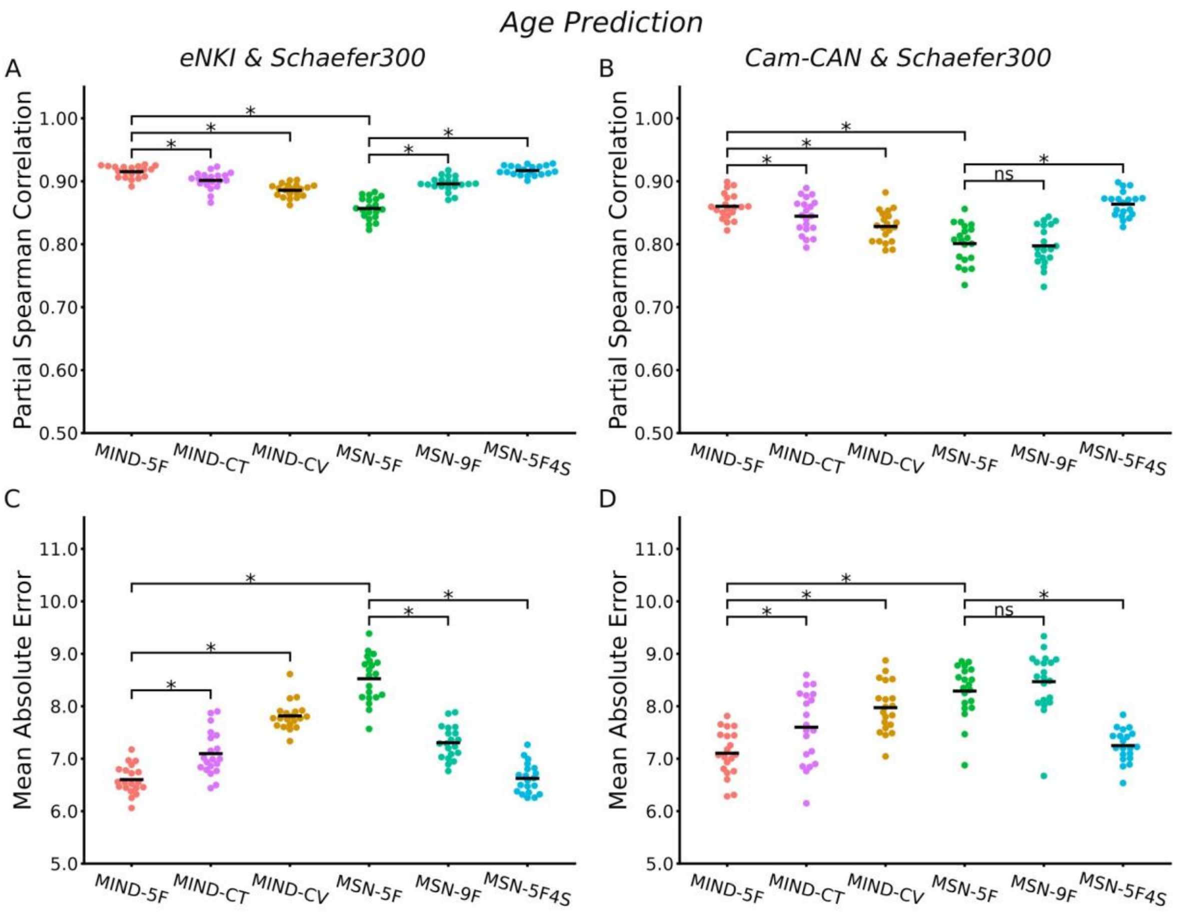

Figure S15. Age prediction performance of models trained on networks derived by MIND-5F, MSN-5F, and their variants using the Schaefer300 atlas in (A, C) the eNKI dataset and (B, D) the Cam-CAN dataset. A 5-fold cross-validation procedure was repeated four times to yield 20 accuracy estimates, each represented by a point in the scatter plot. In each fold, prediction accuracy was quantified by (A, B) the partial Spearman correlation and (C, D) the mean absolute error between predicted and true age. Statistical differences in prediction accuracy between networks derived by MIND-5F and MSN-5F, as well as between networks derived by MIND-5F/MSN-5F and their respective variants, were assessed using paired t-tests. Asterisks indicate statistical significance at an alpha level of 0.05, while “ns” denotes non-significance.

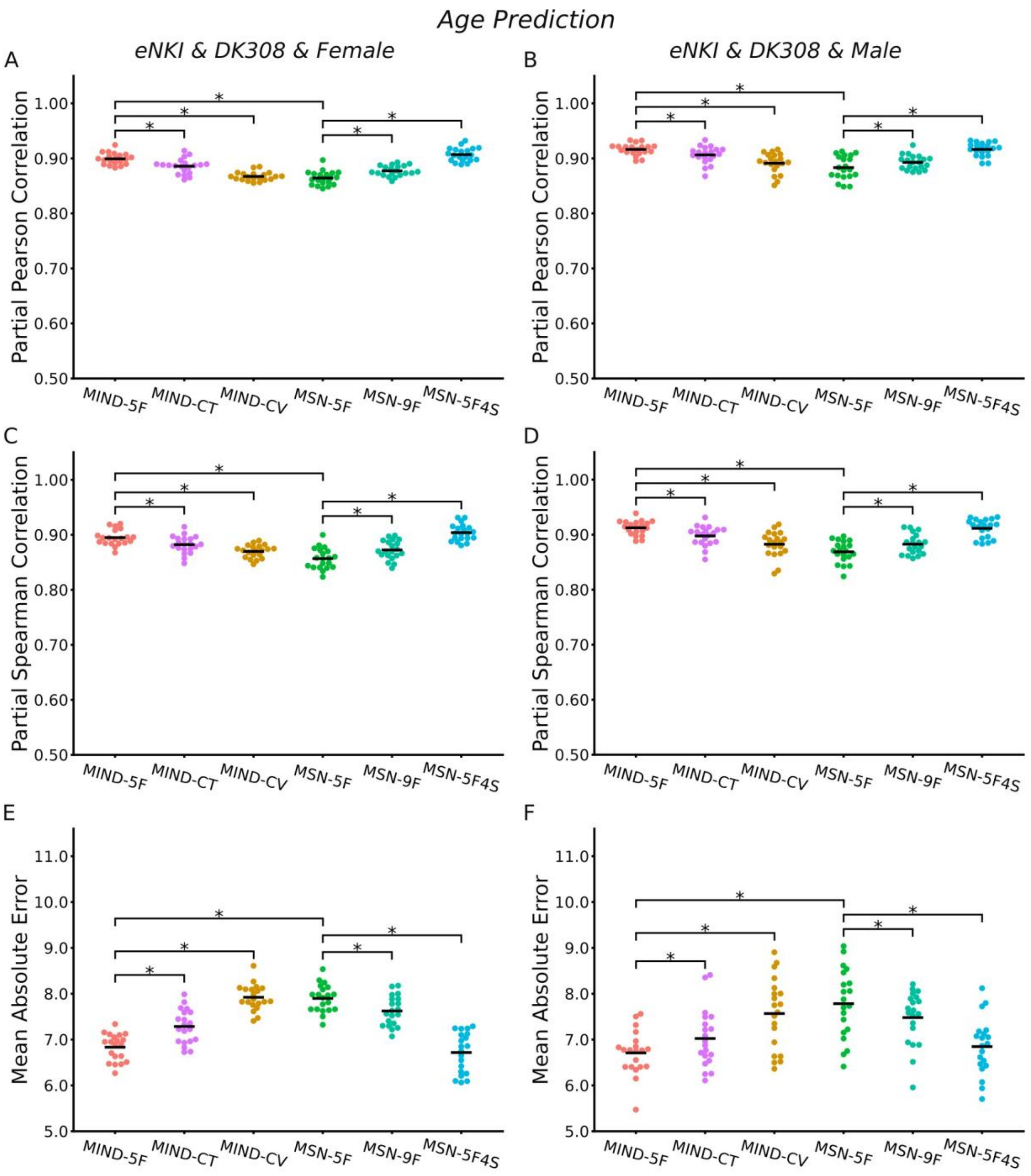

Figure S16. Sex-stratified age prediction performance for models trained on networks derived by MIND-5F, MSN-5F, and their variants using the DK308 atlas in the eNKI dataset. Panels A, C, E correspond to female participants; panels B, D, F correspond to male participants. A 5-fold cross-validation procedure was repeated four times to yield 20 accuracy estimates, each represented by a point in the scatter plot. In each fold, prediction accuracy was quantified by (A, B) the partial Pearson correlation, (C, D) the partial Spearman correlation and (E, F) the mean absolute error between predicted and true age. Statistical differences in prediction accuracy between networks derived by MIND-5F and MSN-5F, as well as between networks derived by MIND-5F/MSN-5F and their respective variants, were assessed using paired t-tests. Asterisks indicate statistical significance at an alpha level of 0.05, while "ns" denotes non-significance.

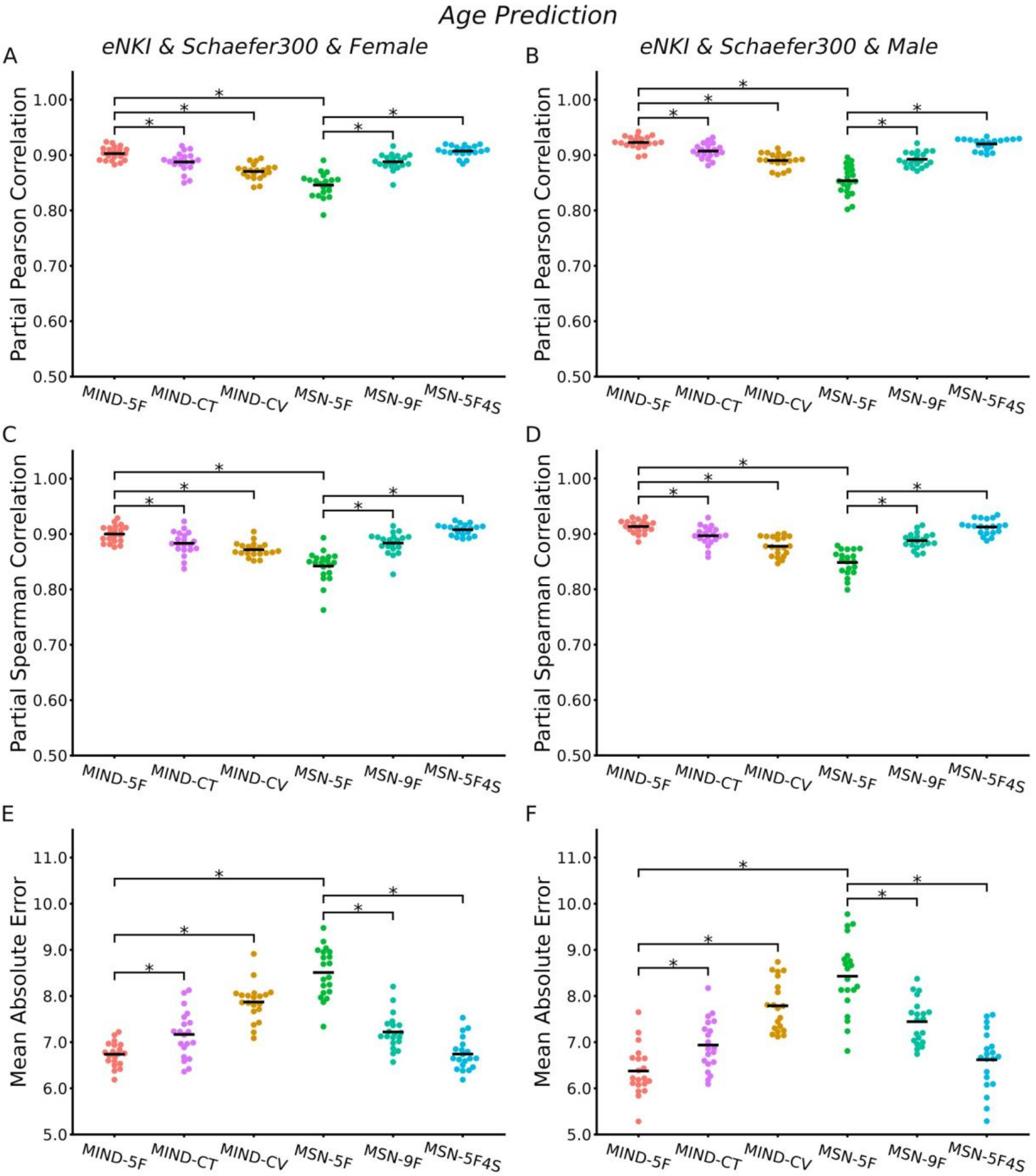

Figure S17. Sex-stratified age prediction performance for models trained on networks derived by MIND-5F, MSN-5F, and their variants using the Schaefer300 atlas in the eNKI dataset. Panels A, C, E correspond to female participants; panels B, D, F correspond to male participants. A 5-fold cross-validation procedure was repeated four times to yield 20 accuracy estimates, each represented by a point in the scatter plot. In each fold, prediction accuracy was quantified by (A, B) the partial Pearson correlation, (C, D) the partial Spearman correlation and (E, F) the mean absolute error between predicted and true age. Statistical differences in prediction accuracy between networks derived by MIND-5F and MSN-5F, as well as between networks derived by MIND-5F/MSN-5F and their respective variants, were assessed using paired t-tests. Asterisks indicate statistical significance at an alpha level of 0.05, while “ns” denotes non-significance.

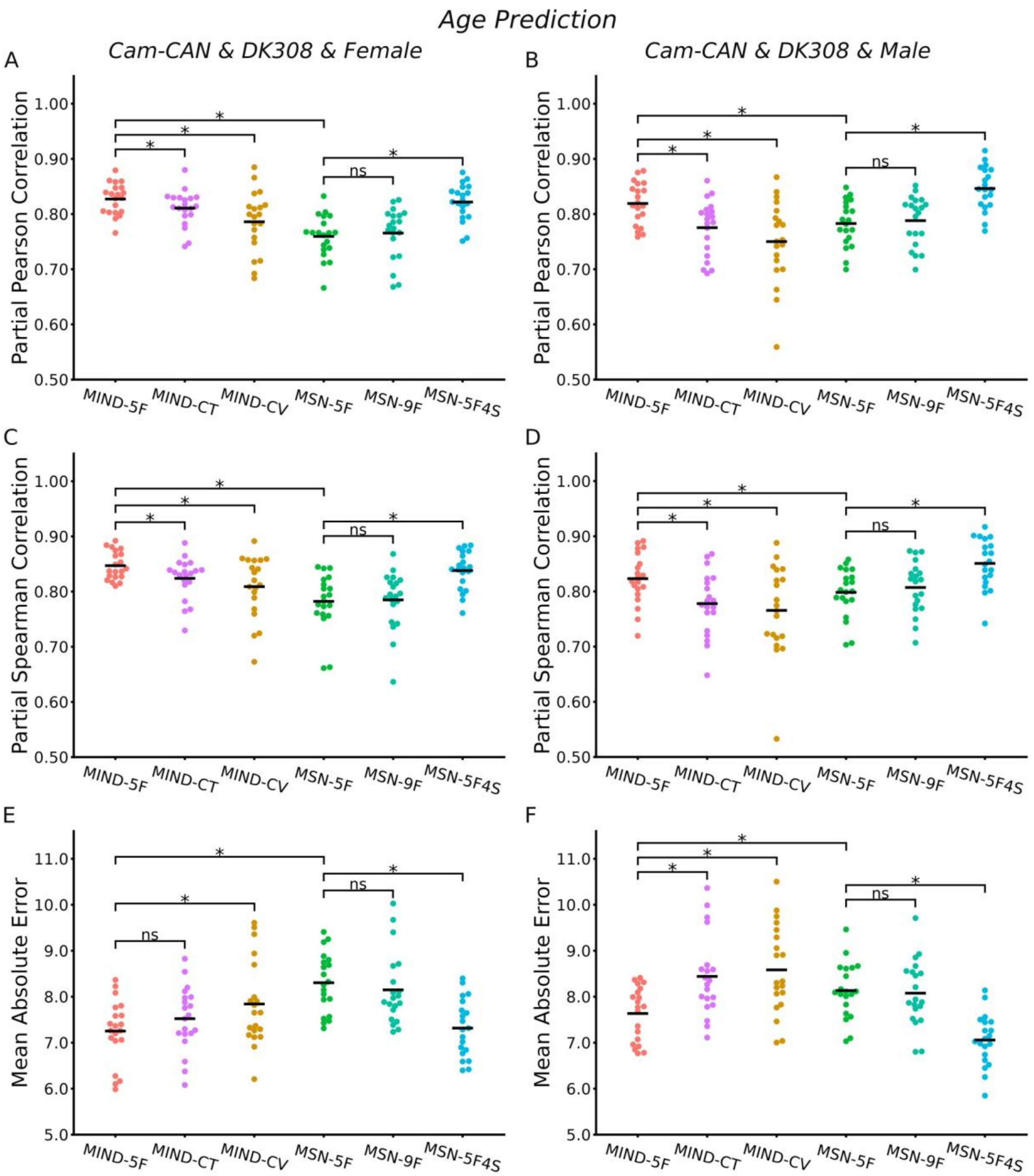

Figure S18. Sex-stratified age prediction performance for models trained on networks derived by MIND-5F, MSN-5F, and their variants using the DK308 atlas in the Cam-CAN dataset. Panels A, C, E correspond to female participants; panels B, D, F correspond to male participants. A 5-fold cross-validation procedure was repeated four times to yield 20 accuracy estimates, each represented by a point in the scatter plot. In each fold, prediction accuracy was quantified by (A, B) the partial Pearson correlation, (C, D) the partial Spearman correlation and (E, F) the mean absolute error between predicted and true age. Statistical differences in prediction accuracy between networks derived by MIND-5F and MSN-5F, as well as between networks derived by MIND-5F/MSN-5F and their respective variants, were assessed using paired t-tests. Asterisks indicate statistical significance at an alpha level of 0.05, while “ns” denotes non-significance.

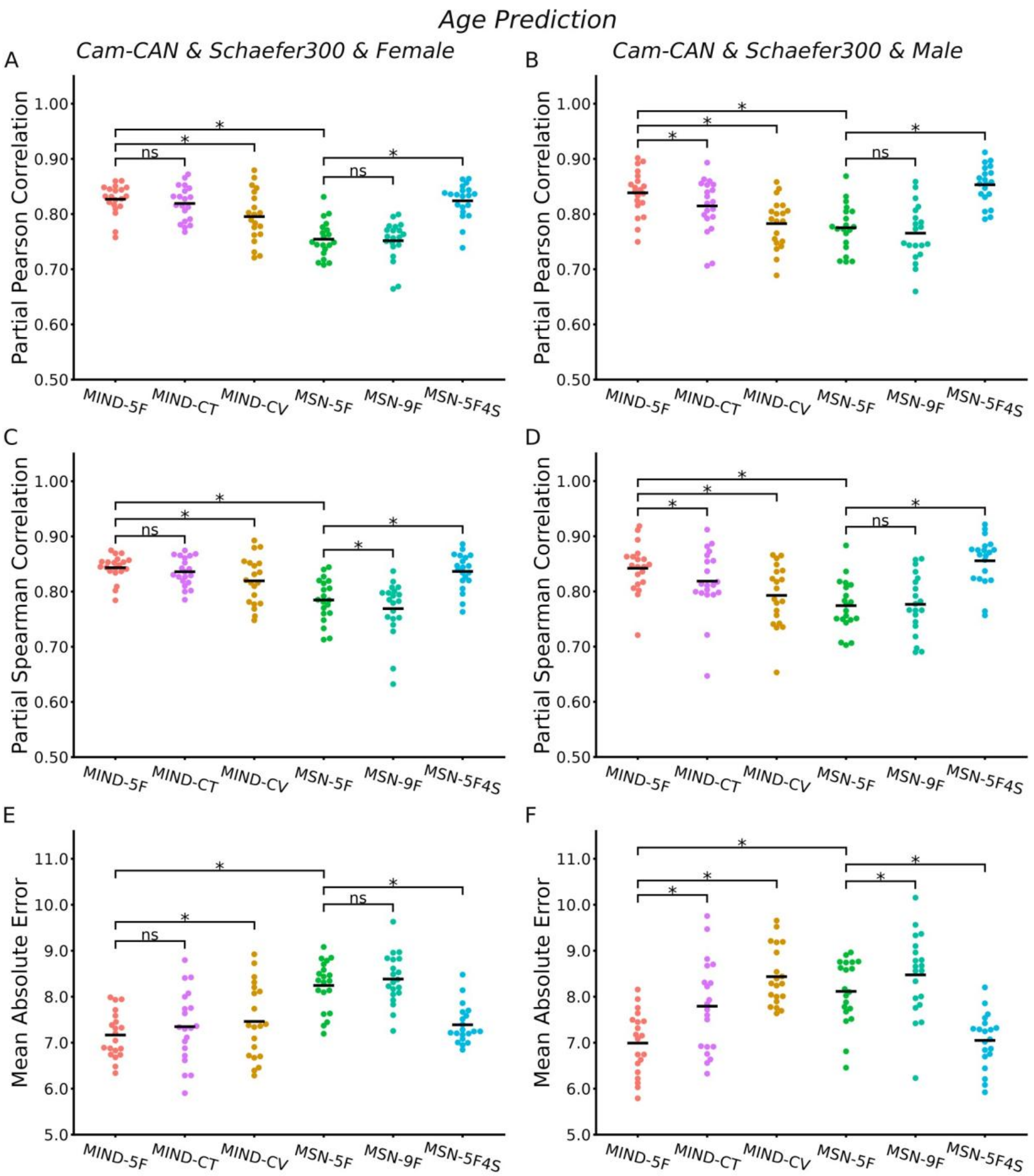

Figure S19. Sex-stratified age prediction performance for models trained on networks derived by MIND-5F, MSN-5F, and their variants using the Schaefer300 atlas in the Cam-CAN dataset. Panels A, C, E correspond to female participants; panels B, D, F correspond to male participants. A 5-fold cross-validation procedure was repeated four times to yield 20 accuracy estimates, each represented by a point in the scatter plot. In each fold, prediction accuracy was quantified by (A, B) the partial Pearson correlation, (C, D) the partial Spearman correlation and (E, F) the mean absolute error between predicted and true age. Statistical differences in prediction accuracy between networks derived by MIND-5F and MSN-5F, as well as between networks derived by MIND-5F/MSN-5F and their respective variants, were assessed using paired t-tests. Asterisks indicate statistical significance at an alpha level of 0.05, while “ns” denotes non-significance.
